# Necl-4/CADM4 regulates GABAergic synaptic strength on GABAergic inhibitory neurons via ErbB4 activation and prevents neuronal impairments

**DOI:** 10.1007/s12035-025-05230-8

**Published:** 2025-07-29

**Authors:** Ryouhei Komaki, Hajime Shiotani, Toshihiko Kuriu, Takeshi Kameyama, Muneaki Miyata, Shin Kedashiro, Kimitaka Katanazaka, Shota Nishii, Norio Chihara, Riki Matsumoto, Michinori Koebis, Atsu Aiba, Kiyohito Mizutani, Yoshimi Takai

**Affiliations:** 1 Division of Pathogenetic Signaling, Department of Psychiatry, Kobe University Graduate School of Medicine, Kobe, Hyogo 650-0047 Japan; 2 Division of Neurology, Kobe University Graduate School of Medicine, Kobe, Hyogo 650-0017 Japan; 3 Division of Pathogenetic Signaling, Institute of Advanced Medical Sciences, Tokushima University, Tokushima, Tokushima, 770-8503 Japan; 4 Center for Medical Research and Development, Osaka Medical and Pharmaceutical University, Takatsuki, Osaka 569-8686 Japan; 5 Clinical Research Center, Osaka Medical and Pharmaceutical University Hospital, Takatsuki, Osaka 569-8686 Japan; 6 Department of Neurology, Kyoto University Graduate School of Medicine, Kyoto, Kyoto, 606-8507 Japan; 7 Section of Animal Research and Laboratory of Animal Resources, Center for Disease Biology and Integrative Medicine, Graduate School of Medicine, The University of Tokyo, Tokyo, 113-0033 Japan

**Keywords:** Neuronal impairments, Inhibitory neuron, GABAergic synapses, ErbB4, GABA receptor, Necl-4/CADM4

## Abstract

Neural networks comprise excitatory and inhibitory neurons, linked through excitatory and inhibitory synapses. Synaptic excitation/inhibition balance is controlled for brain development and functions, and its dysregulations are implicated in aging-dependent neuronal impairments. Here, we found that Necl-4/CADM4, an immunoglobulin superfamily cell adhesion molecule, is expressed in γ-aminobutyric acidergic (GABAergic) inhibitory neurons and localizes at GABAergic synapses on inhibitory neurons in cultured hippocampal neurons and the mouse hippocampus. *Necl-4* genetic ablation induced neuronal loss with synaptic degenerations in the hippocampus, and cultured *Necl-4*-knockout (KO) hippocampal neurons were more susceptible to death. Prior to the neuronal death, the *Necl-4*-KO hippocampal neurons showed an increase in GABAergic synapse density on inhibitory neurons and in synaptic molecules at GABAergic synapses on inhibitory neurons, which were regulated by ErbB4 activation. Furthermore, electrophysiological analysis revealed that *Necl-4* genetic ablation enhanced GABAergic synaptic currents on inhibitory neurons and induced high-frequency firing in dissociated hippocampal cultures composed of glutamatergic excitatory neurons and inhibitory neurons, contributing to excitotoxicity-mediated neuronal death. Thus, Necl-4 regulates GABAergic synaptic strength on inhibitory neurons via ErbB4 activation and prevents neuronal impairments.

**Supplementary Information:**

The online version contains supplementary material available at 10.1007/s12035-025-05230-8.

## Introduction

Neural networks are essential for brain development and functions, but they become impaired with age, leading to the development of various neurological diseases, such as schizophrenia, autism spectrum disorder, Alzheimer’s disease (AD), and epilepsy, in certain populations [1–6]. However, the mechanisms for these aging-dependent neural network impairments or neurological disease onset and development have not been fully understood.

The main pathways of neural networks are formed by excitatory neurons via excitatory synapses. Excitatory neurons are regulated by local neural networks formed by inhibitory neurons, acting via inhibitory synapses in the brain [7, 8]. The major neurotransmitters for excitatory and inhibitory neurons are glutamate and γ-aminobutyric acid (GABA), respectively [9]. In this study, glutamatergic excitatory neurons and γ-aminobutyric acidergic (GABAergic) inhibitory neurons are referred to simply as excitatory neurons and inhibitory neurons, respectively. Accordingly, four types of synapses can be distinguished between excitatory and inhibitory neurons: *E* → *E* synapses (excitatory glutamatergic synapses on excitatory neurons), *E* → *I* synapses (excitatory glutamatergic synapses on inhibitory neurons), *I* → *E* synapses (inhibitory GABAergic synapses on excitatory neurons), and *I* → *I* synapses (inhibitory GABAergic synapses on inhibitory neurons).

In the hippocampus, excitatory neurons in the dentate gyrus (DG), the CA3 region (CA3), and the CA1 region (CA1) are interconnected through *E* → *E* synapses to form the tri-synaptic circuit [7]. In the DG, major excitatory neurons are granule cells, and in the CA3 and CA1, major excitatory neurons are pyramidal cells [10, 11]. Various types of inhibitory neurons have also been identified in these regions, including basket cells, axo-axonic cells (also called chandelier-type cells), and bistratified cells [12]. However, inhibitory neurons account for only 10–15% of all neurons in the hippocampus [13].

Both glutamatergic and GABAergic synapses exhibit synaptic transmission and plasticity, which are considered the molecular basis for learning and memory [14, 15]. In addition, synaptic excitation/inhibition (E/I) balance is critical for these functions and for neural development [8, 16, 17], and E/I dysfunctions are implicated in aging-dependent neuronal impairments leading to various neurological diseases [1–6].

E/I balance is well controlled by four types of synapses. Glutamate release from* E* → *E* and *E* → *I* synapses is suppressed by GABA released from *I* → *E* synapses, the activity of which is induced by glutamate released from *E* → *I* synapses. GABA release from *I* → *E* synapses is suppressed by GABA released from *I* → *I* synapses [18]. These four types of synapses are closely linked in function, with each type influencing the function and organization of the others, which complicates the process of elucidating the specific function of each synapse. Among the four types of synapses, the regulatory mechanisms of *E* → *E* synapses have been largely clarified through studies on molecules specifically localized at *E* → *E* synapses and their morphological characteristics. Additionally, their involvement in neuronal impairments has also been investigated. However, the regulatory mechanisms of the other types of synapses and their implications for neuronal impairments are still obscure. In particular, the regulatory mechanisms of *I* → *I* synapses are the least well understood, and their functions in controlling the E/I balance of neural network activity remain entirely undetermined [3]. This is because GABA_A_ receptor (GABA_A_R) antagonists, such as gabazine and bicuculline, are active at GABA_A_Rs on both *I* → *E* and *I* → *I* synapses, and specific antagonists or agonists for GABA_A_Rs at each synapse have not been developed, nor have any molecules specifically expressed at each synapse type been identified [19–23]. In neural circuits, activation of *I* → *E* synapses directly inhibits hyperexcitability in excitatory neurons, preventing neuronal damage caused by excitotoxicity. In contrast, *I* → *I* synapses have the opposite effect. Therefore, elucidating the regulatory mechanisms of E/I balance and understanding the disorders caused by dysfunctions of these four types of synapses are crucial for comprehending both the physiological and pathological processes of synaptic function.

Here we report that in the mouse hippocampus and cultured hippocampal neurons, the Necl-4/CADM4 cell adhesion molecule is expressed in inhibitory neurons, localizes predominantly at *I* → *I* synapses, regulates the E/I balance of the entire neural network activity, and modulates GABAergic synaptic strength on inhibitory neurons via ErbB4 and GABA_A_R to prevent excessive glutamate release-induced synaptic degenerations and neuronal death caused by excitotoxicity.

## Materials and Methods

### Animals

C57BL/6 mice were purchased from CLEA Japan. B6.129S2-*Emx1*^*tm1(cre)Krj*^/J [24] and B6.Cg-*Gt(ROSA)26Sor*^*tm9(CAG−tdTomato)Hze*^/J [25] were purchased from the Jackson Laboratory and crossed to generate Emx1-Cre;tdTomato mice. They were maintained under a 12/12 light–dark cycle. Postnatal day 0 (P0) was defined as the day of birth.

### Generation of *Necl-4*-KO mice

*Necl-4*-KO mice were generated using the CRISPR/Cas9 system, as described previously, with slight modifications [26]. Briefly, a single-guide RNA (sgRNA) targeting exon 2 of the *Necl-4* gene was selected using CRISPRdirect (http://crispr.dbcls.jp/). The protospacer sequence of the sgRNA was 5’-TGGGTTCTGAATGACGACTA-3’. Cas9/gRNA ribonucleoprotein complex was delivered into fertilized C57BL/6N embryos by electroporation. Embryos were washed three times with Opti-MEM (Thermo Fisher Scientific) supplemented with 0.1% polyvinyl alcohol (PVA) and once with 0.1% PVA-Opti-MEM I, containing Cas9 protein (50 ng/µL, Takara Bio) and the sgRNA (25 ng/µL, Fasmac), placed in a line between two electrodes (LF501PT1-10, BEX) filled with Cas9/gRNA containing 0.1% PVA-Opti-MEM I (total volume, 5 µL), and then electroporation was performed. Voltage pulses (30 V, 3 ms) were applied seven times at intervals of 100 ms using an electroporator CUY21EDIT II (BEX). After electroporation, embryos were immediately collected from the electrode chamber and subjected to three washes with modified Whitten’s medium. Electroporated embryos were transferred into oviducts of 0.5-day-post-coitum ICR recipients (Charles River Laboratories JAPAN). Mice were genotyped by PCR using DNA from tail biopsies. Primer sequences for genotyping were as follows: forward, 5’-GGACGGGACAGGAAGTACAG-3’; and reverse, 5’-TGGTGCCATTGAAAAAGAGG-3’. The PCR reaction consisted of an initial denaturation step of 2 min at 94ºC, followed by 40 cycles of 20 s at 98ºC and 2 min at 68ºC. The primer pair forward and reverse gave a 144 bp for a wild-type (WT) allele and a 134 bp for a mutant allele. They were kept on a C57BL/6 background, and control and mutant mice were prepared from the same litter.

### Cell Culture

Cultured mouse hippocampal neurons obtained from male and female pups were prepared as described previously [27]. In brief, hippocampal neurons dissociated with trypsin were plated at a density of 1.5 × 10^4^ cells/cm^2^ on poly-L-lysine-coated coverslips in Minimum Essential Medium (Thermo Fisher Scientific) with 10% fetal bovine serum and cultured at 37ºC in a humidified 5% CO_2_ incubator. After a 3 h incubation, all medium was replaced with Neurobasal-A Medium (Thermo Fisher Scientific) containing B-27 Supplement (Thermo Fisher Scientific) and GlutaMAX (Thermo Fisher Scientific). Cytosine arabinoside (1 µM) was added to the cultures after 2 days to inhibit glial proliferation. HEK293E cells (derived from a female fetus) for culture were prepared as described previously [28]. Human mammary ductal carcinoma T47D cells (derived from female human) were purchased from ATCC and maintained in RPMI-1640 medium (D-glucose, HEPES, L-glutamine, and phenol red included) supplemented with 10% fetal bovine serum and 10 µg/mL insulin (Fujifilm), and cultured at 37°C in 5% CO_2_, as described previously [29]. For stable expression of Necl-4, pCAGIPuro-FLAG-Necl-4 was introduced with Lipofectamine 3000 (Invitrogen) according to the manufacturer’s protocol.

### Antibodies (Abs) and Reagents

Abs listed below were purchased from commercial sources. Mouse anti-actin monoclonal Ab (mAb) (Merck Millipore, MAB1501), rabbit anti-Calretinin polyclonal Ab (pAb) (Swant, CR 7697), rat anti-Ctip2 mAb (Abcam, ab18465), guinea pig anti-EAAT1 pAb (FRONTIER INSTITUTE, GLAST-GP-Af1000), guinea pig anti-EAAT2 pAb (Merck Millipore, AB1783), guinea pig anti-EAAT2 pAb (FRONTIER INSTITUTE, GLT1-GP-Af810), goat anti-EAAT2 pAb (FRONTIER INSTITUTE, GLT1-Go-Af760), rabbit anti-ErbB4 mAb (Cell Signaling Technology, #4795), rabbit anti-ErbB4/HER4 (phospho Y1284) pAb (Abcam, ab61059), mouse anti-FLAG M2 mAb (Sigma Aldrich, F3165), guinea pig anti-GABA_A_ receptor (GABA_A_R) α1 pAb (FRONTIER INSTITUTE, GABAARa1-GP-Af440), mouse anti-GABA_A_R β2/3 mAb (Merck Millipore, MAB341), mouse anti-GAD67 mAb (Merck Millipore, MAB5406), biotin-conjugated mouse anti-GAD67 mAb (Sigma Aldrich, MAB5406B), rabbit anti-GAT-3 pAb (FRONTIER INSTITUTE, GAT3(590–627)-Rb-Af440), mouse anti-gephyrin mAb (Synaptic Systems, 147 011), guinea pig anti-gephyrin mAb (Synaptic Systems, 147 318), chicken anti-GFAP pAb (Abcam, ab134436), rabbit anti-GFP pAb (MBL, 598), mouse anti-homer-1 mAb (Synaptic Systems, 160 011), chicken anti-MAP2 pAb (Abcam, ab5392), rat anti-MBP mAb (Novus Biologicals, NB600-717), mouse anti-Necl-4 mAb (NeuroMab, 75–247), goat anti-PV pAb (FRONTIER INSTITUTE, PV-Go-Af460), Cy3-conjugated anti-RFP single domain Ab (Synaptic Systems, N0404-SC3-L), mouse anti-tau-1 mAb (Merck Millipore, MAB3420), guinea pig anti-VGAT pAb (FRONTIER INSTITUTE, VGAT-GP-Af1000), rabbit anti-VGAT pAb (FRONTIER INSTITUTE, VGAT-Rb-Af500), guinea pig anti-VGLUT1 pAb (Merck Millipore, AB5905), and rabbit anti-VGLUT1 pAb (FRONTIER INSTITUTE, VGluT1-Rb-Af500). Primary Abs were visualized using goat or donkey fluorochrome-conjugated secondary Abs. The fluorochromes, Alexa Fluor™ 405, 488, 555, 568, and 647 (Thermo Fisher Scientific) were used. Biotin-conjugated Abs were visualized using Streptavidin-conjugated Alexa Fluor™ 750 (Thermo Fisher Scientific). (+)-MK-801 maleate, a non-competitive NMDA antagonist, was purchased from Abcam (ab120027). 6-Cyano-7-nitroquinoxaline-2,3-dione (CNQX) was purchased from Tocris Bioscience (#0190). Neuregulin (NRG1) was purchased from Sigma-Aldrich (H0786). Afatinib was purchased from Selleck Chemicals (S1011).

### Plasmid Construction

cDNA for human *ERBB4* were kindly provided by Dr. Shigeki Higashiyama (Ehime University, Japan). pCAGIPuro-FLAG-Necl-4 and pEGFP-N3-ErbB4 were constructed as described previously [28, 30].

### Immunofluorescence Microscopy

Immunofluorescence microscopy of cultured cells was performed as described previously [27]. In brief, cultured hippocampal neurons were fixed in 2% paraformaldehyde (PFA) in 0.1 M phosphate buffer at 37ºC for 10 min. Fixed cells were permeabilized with 0.25% Triton X-100 in phosphate-buffered saline (PBS) at room temperature for 10 min. Then, cells were incubated with 1% bovine serum albumin (BSA) in either PBS or Tris-buffered saline (TBS) containing 1 mM CaCl_2_, followed by 10% goat serum containing 0.25% Triton X-100 for 30 min each. Next, cells were incubated with primary Abs in either PBS containing 3% goat serum and 0.25% Triton X-100, TBS containing 3% goat serum, 0.25% Triton X-100, and 1 mM CaCl_2_, or Can Get Signal immunostain Immunoreaction Enhancer Solution (Toyobo) at 4ºC overnight. After three 5-min washes in either PBS or TBS containing 1 mM CaCl_2_, cells were incubated with fluorochrome-conjugated secondary Abs (Thermo Fisher Scientific) at room temperature for 2 h, followed by three 5-min washes in either PBS or TBS containing 1 mM CaCl_2_ at room temperature. Samples were then mounted in FluorSave reagent (Merck Millipore). Immunofluorescence image analysis was performed on a BZ-X710 All-in-One Fluorescence Microscope (KEYENCE) using a Plan Apo λ 100 ×/1.45 numerical aperture oil immersion objective lens (Nikon) with a resolution of 1920 × 1440 pixels. Images were acquired as a z-stack (2–8 optical sections, 0.5-μm step size), and maximum intensity projections were created from the stacks if necessary. Structured illumination image analysis was performed on an LSM 780/ELYRA PS.1 microscope (Carl Zeiss) using a Plan Apochromat 100 ×/1.46 numerical aperture oil immersion objective lens (Carl Zeiss). Images were then reconstructed using ZEN software (Carl Zeiss) based on the structured illumination algorithm.

### Quantification of Necl-4 Expression and Localization In Vitro

Quantification of Necl-4 expression in excitatory and inhibitory neurons was performed by calculating Necl-4 signal intensity in the regions of interest (ROIs) within the cell bodies after classification of excitatory and inhibitory neurons based on the GAD67 fluorescence signal intensity. Because there are various subgroups of inhibitory neurons, the inhibitory neurons were selected based on their morphology, as represented in Fig. S1. The characteristics of the selected inhibitory neurons were as follows: their cell bodies were approximately twice the size of those of excitatory neurons, they extended fewer dendrites and branches compared to excitatory neurons, and their GAD67 fluorescence signal intensity was stronger than average. Necl-4 localization along dendrites and axons was assessed by measuring the Necl-4 signal intensity in ROIs corresponding to dendrites (MAP2-positive and tau-1-negative neuronal processes) and axons (MAP2-negative and tau-1-positive neuronal processes) after classification of excitatory and inhibitory neurons. Necl-4 signal intensity at each synapse was measured in ROIs of VGUT1-positive puncta for *E* → *E* and *E* → *I* synapses and VGAT-positive puncta for *I* → *E* and *I* → *I* synapses after classification of excitatory and inhibitory neurons. To compare these four types of synapses, neurons were immunostained for Necl-4, VGLUT1, VGAT, GAD67, and MAP2. Necl-4 positivity at each synapse was quantified using the following method. Each image of VGLUT1, VGAT, or Necl-4 was binarized based on its signal intensity by setting a threshold value (mean + 2SD) for each region within 4.5 μm of the MAP2-positive dendrites. From the binarized images, puncta larger than 0.04 μm^2^ and less than 2 μm^2^ were selected using the “Analyze Particle” function of ImageJ. When the Necl-4 puncta were overlapped with the VGLUT1 puncta by at least one pixel, they were judged to be colocalized at *E* → *E* or *I* → *E* synapses, while when the Necl-4 puncta were overlapped with the VGAT puncta by at least one pixel, they were judged to be colocalized at *I* → *E* or *I* → *I* synapses. The percentage of Necl-4-positive synapses was calculated by dividing the number of Necl-4-positive *E* → *I* or *I* → *E* synapses by the total number of *E* → *I* or* I* → *I* synapses, respectively.

### Quantification of Excitatory and Inhibitory Neuron Densities In Vitro

Quantification of excitatory and inhibitory neuron densities in vitro was performed on six to ten randomly selected fields of 4 mm^2^ using ImageJ. At least three independent experiments were conducted. The number of MAP2-positive cells in each subfield was manually counted.

### Quantification of Synapse Densities and Synaptic Marker Signal Intensities

For quantification, maximum signal intensity projection images were created from 2–8 optical images collected at 0.5-μm steps along the z-axis with a BZ-X710 All-in-One Fluorescence Microscope (KEYENCE) under the same conditions for both WT and *Necl-4*-KO neurons. Second branches of MAP2-positive dendrites within 90 μm of the cell body of neurons were randomly selected. Of these, a 20-μm area that did not intersect other MAP2-positive dendrites was analyzed. Each image of the indicated synaptic marker in each region was binarized based on its signal intensity by setting a threshold value (mean + 2SD) for each. From binarized images, puncta larger than 0.04 μm^2^ were selected using the “Analyze Particle” function of ImageJ. Among these regions, those that were double-positive for the VGLUT1 and homer-1 signals and smaller than 2 μm^2^ were considered excitatory synapses (*E* → *E* or *E* → *I* synapses), and those that were double-positive for the VGAT and gephyrin signals and smaller than 2 μm^2^ in size were considered inhibitory synapses (*I* → *E* or *I* → *I* synapses). The number of synapses was then automatically counted to calculate synapse densities. Mean fluorescence signal intensities of the indicated synaptic markers, such as gephyrin, VGAT, homer-1, VGLUT1, GABA_A_R α1 subunit, and GABA_A_R β2/3 subunits, were measured in the initial maximum signal intensity projection images and adjusted by min–max normalization.

### Immunohistochemistry

Immunofluorescence microscopy of brain sections was performed as described previously [27]. In brief, WT or *Necl-4*-KO male mice at postnatal day 56 (P56) or 19 weeks of age (19 wk) were deeply anesthetized and transcardially perfused at room temperature with HBSS containing 10 mM HEPES, 1 mM sodium pyruvate, 4% sucrose, heparin, and protease inhibitor cocktail (cOmplete Mini; Roche Diagnostics), followed by perfusion of 2% PFA in the above-mentioned HBSS-based buffer. After dehydration with 30% sucrose in PBS, whole brains were embedded in OCT compound (Sakura Finetek). Cryostat sections were incubated in HistoVT One antigen retrieval solution (Nacalai Tesque) at 62ºC for 20 min and then incubated with 1% BSA, 10% normal donkey serum, and 0.25% Triton X-100 in PBS at room temperature for 30 min. Sections were stained with the indicated Abs, and then with appropriate fluorochrome-conjugated secondary Abs (1:300). Fluorescence microscopic image analysis was performed on a BZ-X710 All-in-One Fluorescence Microscope (KEYENCE) using a Plan Apochromat 20 ×/0.95 numerical aperture objective lens (Nikon). Images captured on the BZ-X710 were analyzed using BZ-X-Analyzer software (KEYENCE). Confocal image analysis was performed on a C2 confocal laser-scanning microscope (Nikon) using Plan Apochromat 60 ×/1.20 numerical aperture water immersion objective lens (Nikon). Images captured on the C2 were analyzed using NIS Elements acquisition software (Nikon). Structured illumination image analysis was performed on an LSM 780/ELYRA PS.1 microscope (Carl Zeiss) using a Plan Apochromat 100 ×/1.46 numerical aperture oil immersion objective lens (Carl Zeiss). Images were then reconstructed using ZEN software (Carl Zeiss), based on the structured illumination algorithm.

### Quantification of Pyramidal Cells, Granule Cells, Parvalbumin (PV)-Positive Inhibitory Neurons, and Calretinin-Positive Inhibitory Neurons In Vivo

To quantify the number of pyramidal and granule cell nuclei, high-magnification tiling images were obtained by imaging the *stratum pyramidale* of CA1 and DG, respectively. DAPI-positive nuclei were manually counted within 100 × 60 μm regions for pyramidal cells and within 100 × 100 μm regions for granule cells. The number of pyramidal cell nuclei was then calculated by subtracting the total numbers of astrocyte nuclei (Fig. 4a, arrowheads) and inhibitory neuron nuclei (Fig. 4a, arrows) from the number of DAPI-positive nuclei. Astrocytic nuclei were identified based on their smaller nuclear morphology and higher signal intensity than pyramidal cells. Inhibitory neuron nuclei were identified based on their positioning, which is spaced apart from surrounding nuclei, in contrast to the densely positioned nuclei observed in pyramidal cells. The respective numbers of PV-positive and calretinin-positive inhibitory neurons at CA1 were manually counted.

### Electrophysiology

Whole-cell patch-clamp recordings were performed on cultured hippocampal neurons at room temperature, following previously described methods [31, 32]. The neurons in the recording chamber were perfused with an extracellular solution containing 119 mM NaCl, 2.5 mM KCl, 30 mM D-glucose, 2 mM CaCl_2_, 2 mM MgCl_2_, and 25 mM HEPES at pH 7.4. To measure membrane potentials, the electrodes were filled with an internal solution consisting of 125 mM K-methanesulfonate, 6 mM KCl, 2 mM MgCl_2_, 0.6 mM EGTA, 3.2 mM Mg-ATP, 1.2 mM Na-GTP, and 10 mM HEPES at pH 7.4 by KOH. The excitatory neurons analyzed were selected for their characteristic of having dendrites with a relatively high number of branches radiating from the cell body, whereas the inhibitory neurons were selected for having fewer dendrites and branches compared to the excitatory neurons. The membrane potentials were corrected for a liquid junction potential of 9 mV. Resting membrane potentials and action potentials were recorded using the current-clamp configuration. Action potentials were identified as overshoots above 0 mV. Miniature inhibitory postsynaptic currents (mIPSCs) at *I* → *I* synapses were recorded from the inhibitory neurons held at −70 mV in the voltage-clamp configuration. The extracellular solution was supplemented with 1 μM TTX (WAKO), 10 μM CNQX (Tocris), 50 μM D-(-)-2-amino-5-phosphonovaleric acid (Tocris), and 1 μM strychnine (Sigma). For mIPSC measurements, the electrodes were filled with an internal solution containing 65 mM Cs-methanesulfonate, 65 mM CsCl, 1 mM CaCl_2,_ 2 mM MgCl_2_, 11 mM EGTA, 3.2 mM Mg-ATP, and 1.2 mM Na-GTP, 10 mM HEPES at pH 7.4 by CsOH, and the membrane potentials were corrected for the liquid junction potential (7 mV). Under these conditions, mIPSCs were blocked by 10 μM bicuculline (Tocris) or 10 μM GABAzine (SR-95531, Toronto Research Chemicals), and they exhibited a reversal potential near the calculated E_Cl_, confirming their GABAergic profile [33]. To ensure accurate detection of mIPSCs, a threshold of three times the root mean square (RMS) of the background noise was applied. Based on this criterion, cells with background noise exceeding 3.3 pA (RMS) were excluded from the analysis. A fixed detection threshold of 10 pA was uniformly applied across all remaining cells. Each detected event was subsequently verified by visual inspection to exclude artifacts. For each cell, the mean and cumulative distributions of event amplitudes and frequencies were analyzed from events recorded during a 5-min measurement. Membrane potentials and currents were recorded using a MultiClamp 700B amplifier (Axon Instruments). The records were filtered at 2 kHz and acquired at 10 kHz. Series resistance was monitored during the experiments, and cells with a series resistance < 30 MΩ were included in the analysis. Data analysis for resting membrane potentials, action potentials, and mIPSCs was performed using Clampfit 11.2 (Axon Instruments), OriginPro 2023b (OriginLab), and MiniAnalysis 6.0.3 (Synaptosoft).

### Immunoprecipitation and Western Blotting

HEK293E cells were co-transfected with pCAGIPuro-FLAG-Necl-4 and pEGFP-N3-ErbB4 and cultured at 37°C for 48 h. Cells were washed with PBS and lysed with lysis buffer (20 mM Tris–HCl at pH 7.5, 1% Nonidet P-40, 10% glycerol, 150 mM NaCl, 1 mM dithiothreitol (DTT), 1 mM CaCl_2_, 1 mM MgCl_2_, 1 mM 4-(2-aminoethyl) benzenesulfonyl fluoride hydrochloride, protease inhibitor cocktail (cOmplete, EDTA-free, Roche Diagnostics, and Phosphatase Inhibitor Cocktail 2 and 3 (Sigma-Aldrich)). Lysates were rotated for 10 min at 4°C, subjected to centrifugation at 4°C at 12,000 × g for 15 min, and incubated with anti-FLAG mAb conjugated Protein G Dynabeads (Thermo Fisher Scientific). Lysates were rotated at room temperature for 15 min. After the beads were extensively washed with lysis buffer, bound proteins were eluted by heating at 80°C in SDS sample buffer (67 mM Tris–HCl at pH 6.8, 2% SDS, 100 mM DTT, 5% sucrose, and 0.005% bromophenol blue) for 2 min, and subjected to SDS-PAGE, followed by Western blotting. Samples separated on SDS-PAGE were transferred to poly(vinylidene difluoride) membranes (Merck Millipore). After being blocked with Block Ace (KAC) in Tris-buffered saline plus 0.005% Tween 20, membranes were incubated with the indicated Abs. After being washed three times with Tris-buffered saline plus 0.05% Tween 20, membranes were incubated with a horseradish peroxidase (HRP)-conjugated anti-mouse or anti-rabbit IgG Ab. Signals for proteins were visualized by incubation with Immobilon Western Chemiluminescent HRP Substrate (Merck Millipore) and then detected using the ImageQuant LAS4000 (GE Healthcare).

### Assay for ErbB4 Activity

T47D cells were plated at a density of 6 × 10^4^ cells/cm^2^ on dishes and cultured for 3 days. Cells were starved of serum with Dulbecco's Modified Eagle Medium containing 0.5% fatty acid-free BSA for 24 h and then stimulated with 20 ng/mL NRG1 in Opti-MEM (Thermo Fisher Scientific) for 0, 1, 2, 4, and 8 min. Cells were washed with ice-cold PBS and lysed with an EzApply (ATTO). Lysates were sonicated on ice. Protein concentrations were determined using a Pierce™ 660nm Protein Assay kit (Thermo Fisher Scientific). Protein lysates were heated at 94ºC for 2 min and subjected to SDS-PAGE followed by Western blotting.

### Statistical Analysis

Normality was determined using the Shapiro–Wilk test. Based on this outcome, statistical analysis of differences between mean values was performed using either the Mann–Whitney U test or two-tailed, unpaired Student’s *t*-test, as appropriate. Bonferroni correction was applied for multiple comparisons. The percentages of Necl-4-positive puncta at *E* → *I* and *I* → *I* synapses were compared using the chi-square test. Cumulative distributions were compared using the Kolmogorov–Smirnov test. Statistical significance was set at p < 0.05. Data are presented as box plots with median lines or as means ± standard error of the mean.

## Results

### Localization of Necl-4 in Cultured Hippocampal Neurons and in the Hippocampus

**Fig. 1:**
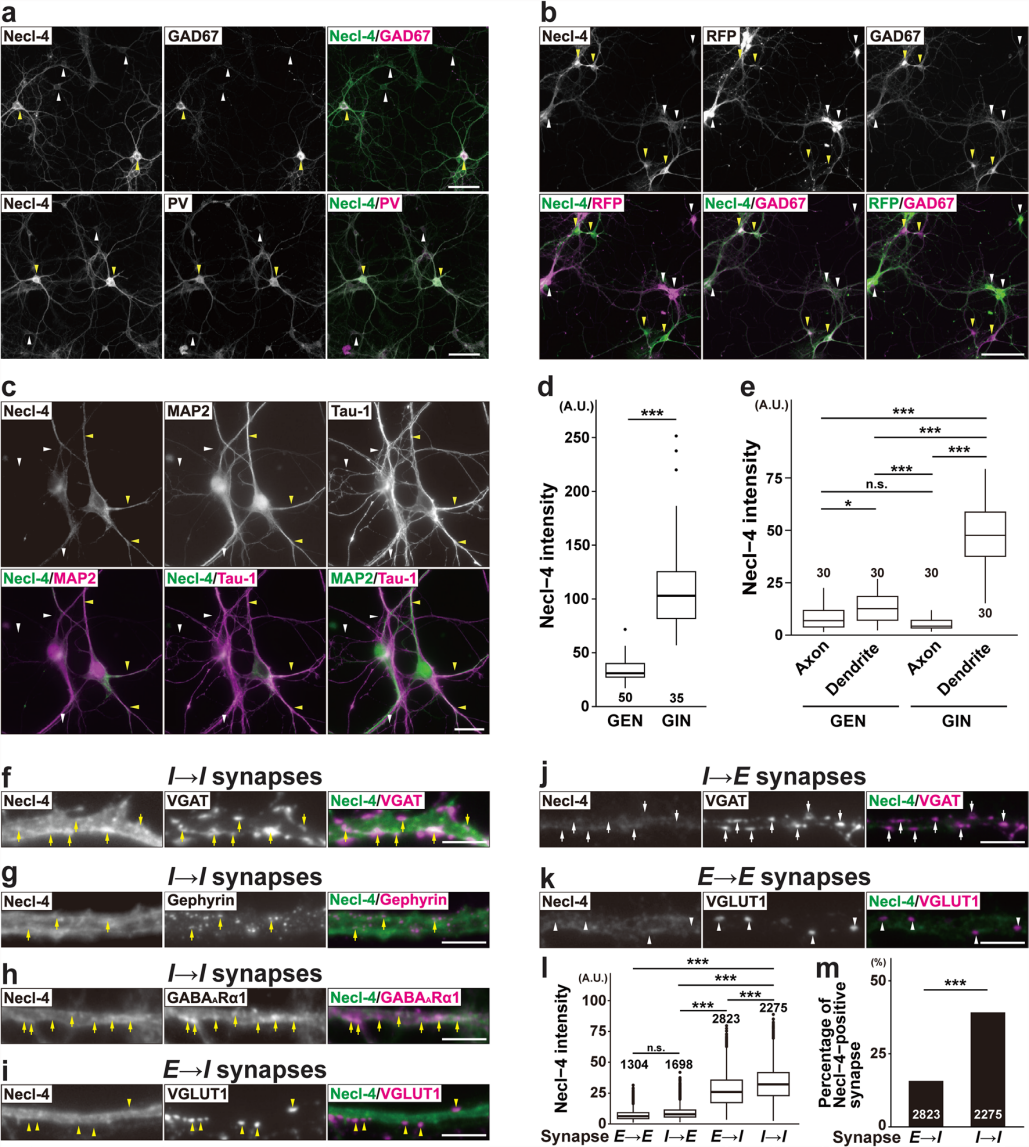
Localization of Necl-4 at synapses in cultured hippocampal neurons. Representative immunofluorescence images using the indicated antibodies (Abs) in respective panels. (a) Cultured wild-type (WT) hippocampal neurons at 21 days in vitro (DIV). Yellow arrowheads, GABAergic inhibitory neurons (GINs). White arrowheads, glutamatergic excitatory neurons (GENs). Bars, 100 μm. (b) Cultured Emx1-Cre;tdTomato neurons at 10 DIV. GENs labeled with tdTomato by Emx1-mediated cre recombination were detected using the red fluorescent protein (RFP) Ab. Yellow arrowheads, GINs. White arrowheads, GENs. Bar, 50 μm. (c) Cultured WT neurons at 10 DIV. Yellow arrowheads, dendrites. White arrowheads, axons. Bar, 20 μm. (d) Quantitative analyses of (a). ***, p < 0.001. Mann-Whitney U test was performed. (e) Quantitative analyses of (c). *, adjusted p < 0.05. ***, adjusted p < 0.001. n.s., not significant. Mann-Whitney U test was performed with a Bonferroni correction. (f–k) Cultured WT neurons at 21 DIV. Yellow arrows, GABAergic synapses on GINs (*I* → *I* synapses). Yellow arrowheads, glutamatergic synapses on GINs (*E* → *I* synapses). White arrows, GABAergic synapses on GENs (*I* → *E* synapses). White arrowheads, glutamatergic synapses on GENs (*E* → *E* synapses). Bars, 5 μm. (l) Quantitative analyses of (f, i, j, k). ***, adjusted p < 0.001. n.s., not significant. Mann-Whitney U test was performed with a Bonferroni correction. (m) Quantitative analyses of (f, i). ***, p < 0.001. Chi-square test was performed. Sample sizes are indicated within each boxplot or barplot. These images are representative of three independent experiments. GABA_A_R α1 subunit (GABA_A_Rα1).

In cultured mouse hippocampal neurons with few astrocytes and no oligodendrocytes, the Necl-4 signal was strongly observed in GAD67-positive inhibitory neurons, including at least PV-positive inhibitory neurons (Fig. 1a, d,Table 1). In contrast, the Necl-4 signal was hardly observed in excitatory neurons that were labeled with tdTomato (a red fluorescent protein) by Emx1-mediated cre recombination (Fig. 1b, d, Table 1). The Necl-4 signal diffusely localized on MAP2-positive processes, but not on tau-1-positive processes **(**Fig. 1c, e**, **Table 1**)**. It predominantly localized near the VGAT, gephyrin, and GABA_A_R α1 subunit signals on dendrites of inhibitory neurons (*I* → *I* synapses) in a punctate manner, and near the VGLUT1 signal on dendrites of inhibitory neurons (*E* → *I* synapses) in a diffuse manner, but not near the VGAT signal on dendrites of excitatory neurons (*I* → *E* synapses) or the VGLUT1 signal on dendrites of excitatory neurons (*E* → *E* synapses) (Fig. 1f–m, Table 1). These results indicate that Necl-4 predominantly accumulates at *I* → *I* synapses and diffusely localizes at *E* → *I* synapses, but not at other synapses. Based on the preferential expression pattern at dendrites in cultured hippocampal neurons, Necl-4 presumably localizes at the postsynaptic sides of these synapses. In these experiments, *E* → *E* and *E* → *I* synapses were identified by co-localization of the VGLUT1 and homer-1 signals, and *I* → *E* and *I* → *I* synapses were identified by co-localization of the VGAT and gephyrin signals after identification of excitatory or inhibitory neurons with the GAD67 signal (Fig. S1a–d, Table 1). The Necl-4 signal was very weak and not observed in a punctate pattern in inhibitory and excitatory neurons derived from *Necl-4*-knockout (KO) mice (Fig. S2a, b).

**Table 1:** Neuronal, astrocytic, and synaptic markers and their characteristic features

Name of molecule	Marker
Calretinin	**GABAergic inhibitory neuron marker**
Ctip2	**Glutamatergic excitatory neuron marker**
EAAT1/2	**Perisynaptic astrocyte process markers at glutamatergic synapses**
Emx1	**Glutamatergic excitatory neuron marker**
GABA_A_R α1 subunit	**Postsynaptic marker for GABAergic synapses**
GAD67	**GABAergic inhibitory neuron marker**
GAT-3	**Perisynaptic astrocyte process markers at GABAergic synapses**
Gephyrin	**Postsynaptic marker for GABAergic synapses**
GFAP	**Astrocyte marker**
Homer-1	**Postsynaptic marker for glutamatergic synapses**
MAP2	**Dendrite marker**
Myelin basic protein (MBP)	**Myelin sheath marker**
Parvalbumin (PV)	**GABAergic inhibitory neuron marker**
Tau-1	**Axon marker**
VGAT	**Presynaptic marker for GABAergic synapses**
VGLUT1	**Presynaptic marker for glutamatergic synapses**

**Fig. 2:**
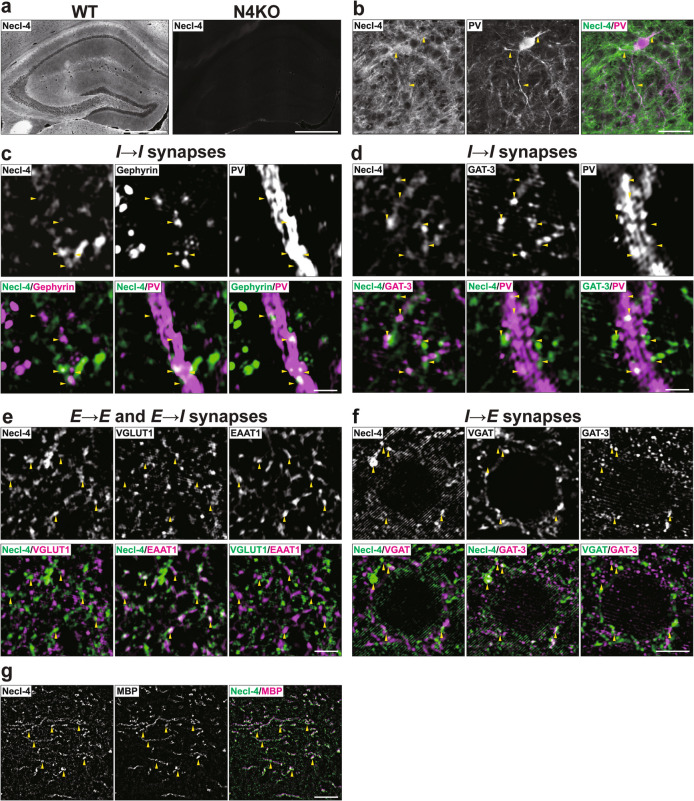
Localization of Necl-4 at synapses, in addition to perisynaptic astrocyte processes and myelin in the hippocampus. Representative images of hippocampi using the indicated antibodies in respective panels. (a) Low-magnification images of hippocampi of wild-type (WT) and *Necl-4*-knockout (N4KO) mice at postnatal day 56 (P56). Bars, 500 μm. (b) High-magnification images of the *stratum lucidum* of CA3 region (CA3) near dentate gyrus (DG) in hippocampi of WT mice at P56. Yellow arrowheads, dendrites of parvalbumin (PV)-positive inhibitory neurons. Bar, 30 μm. (c, d) Super-resolution structured illumination microscopy (SIM) images of the *stratum lucidum* of CA3 near DG in hippocampi of WT mice at P56. Yellow arrowheads, GABAergic synapses on inhibitory neurons (*I* → *I* synapses) on dendrites of PV-positive inhibitory neurons. Bars, 1 μm. (e) SIM images of the *stratum radiatum* of CA1 region (CA1) in hippocampi of WT mice at 19 weeks of age (19 wk). Yellow arrowheads, glutamatergic synapses on excitatory neurons and inhibitory neurons (*E* → *E* and *E* → *I* synapses). Bar, 2 μm. (f) SIM images of a pyramidal cell at the *stratum pyramidale* of CA1 in hippocampi of WT mice at 19 wk. Yellow arrowheads, GABAergic synapses on excitatory neurons (*I* → *E* synapses). Bar, 4 μm. (g) SIM images of the *stratum lucidum* of CA3 near DG in hippocampi of WT mice at P56. Yellow arrowheads, myelin sheath. Bar, 10 μm. These images are representative of three independent experiments.

In hippocampi of WT mice at P56, the Necl-4 signal was observed in many regions, including DG, CA3, and CA1, but not in *Necl-4-*KO mice (Fig. 2a). The Necl-4 signal localized at least on dendrites of PV-positive inhibitory neurons (Fig. 2b). In super-resolution structured illumination microscopy (SIM), the Necl-4 signal localized near the gephyrin signal along PV-positive dendrites and the GAT-3 signal at *I* → *I* synapses (Fig. 2c, d, Table 1). In addition, it localized at the EAAT1-positive perisynaptic astrocyte processes (PAPs) of *E* → *E* and *E* → *I* synapses and the GAT-3-positive PAPs of *I* → *E* synapses, but not at the synapses themselves (Fig. 2e, f, Table 1). It also localized near the myelin basic protein (MBP)-positive oligodendrocytes (Fig. 2g, Table 1), as described previously [34]. These results indicate that Necl-4 localizes at *I* → *I* synapses and PAPs of *E* → *E*, *E* → *I*, *I* → *E*, and *I* → *I* synapses in addition to oligodendrocytes in the hippocampus.

### Neuronal Loss with Synaptic Degenerations in the *Necl-4*-KO Hippocampus

**Fig. 3:**
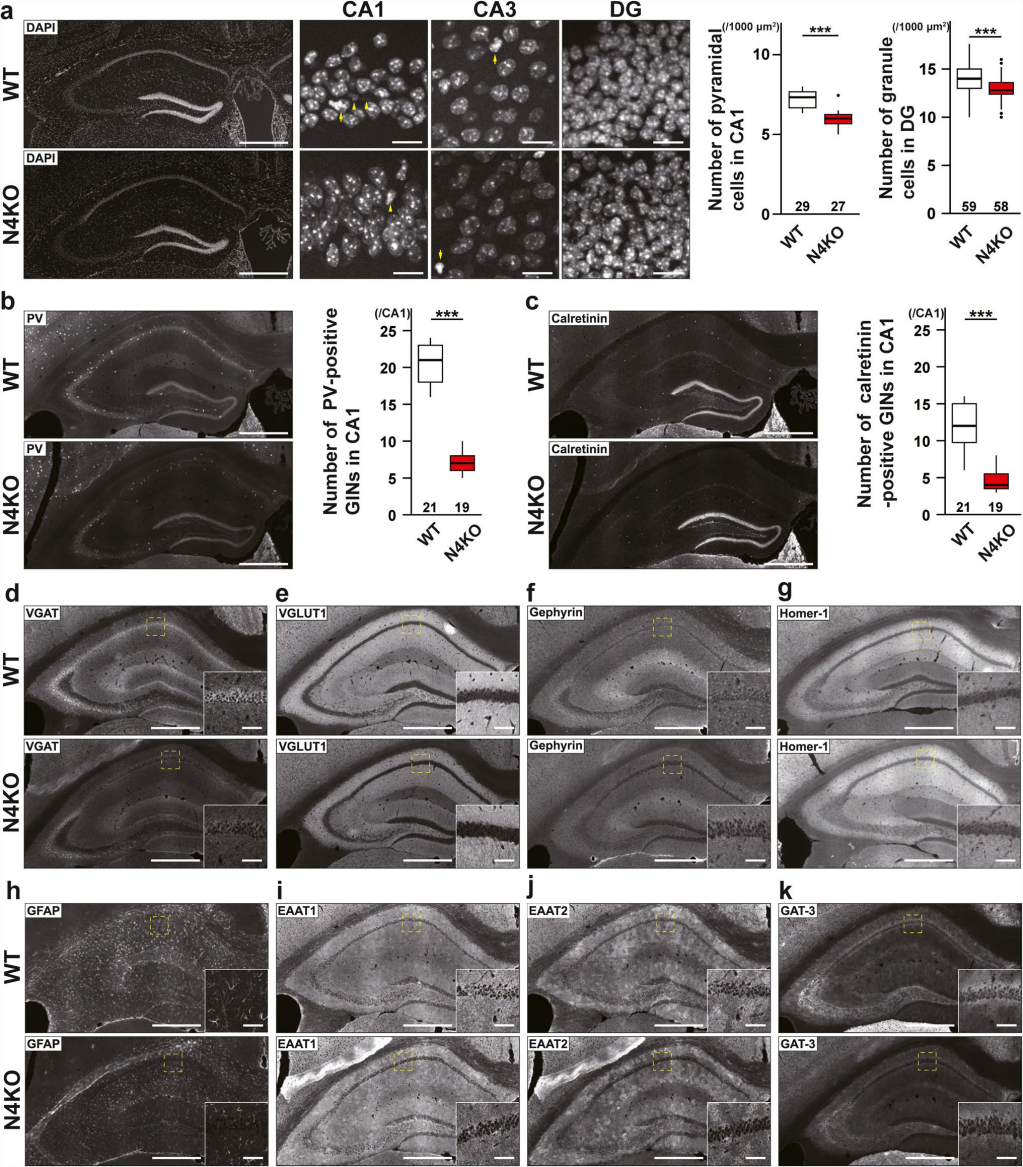
Synaptic degenerations and neuronal loss in the *Necl-4*-knockout (N4KO) hippocampus. Representative images of hippocampi of wild-type (WT) and N4KO mice at 19 weeks of age using the indicated antibodies in respective panels. (a left, b, c) Low-magnification images of hippocampi. Bars, 500 μm. (a center), High-magnification images of subregions of hippocampi. Bars, 10 μm. Yellow arrowheads, nuclei of GABAergic inhibitory neurons (GINs). Yellow arrows, nuclei of astrocytes. (a right) Quantitative analyses are shown. ***, p < 0.001. Mann-Whitney U tests were performed. Sample sizes are indicated within each boxplot. (d–k) Low-magnification images of hippocampi. Bars, 500 μm. Insets, High-magnification images of the boxed region. Bars, 10 μm. These images are representative of three independent experiments.

**Fig. 4:**
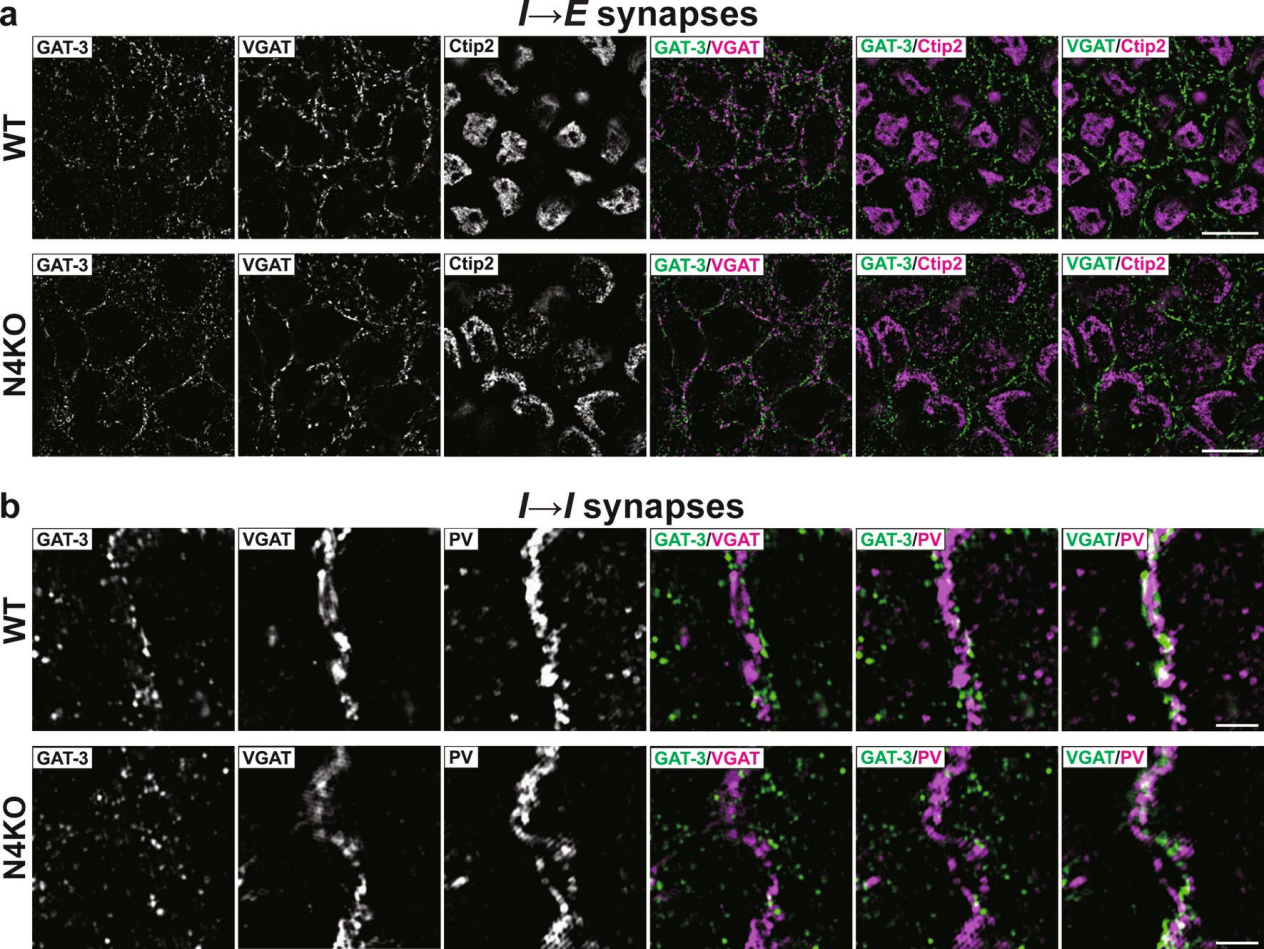
Synaptic degenerations in the *Necl-4*-knockout (N4KO) hippocampus. Representative images of hippocampi of wild-type (WT) and N4KO mice at 19 weeks of age using the indicated antibodies in respective panels. (a) Super-resolution structured illumination microscopy (SIM) images of the *stratum pyramidale* of CA1 region. (b) SIM images of the *stratum lucidum* of CA3 region near dentate gyrus. Bars, 10 μm (a), and 2 μm (b). These images are representative of three independent experiments.

**Fig. 5:**
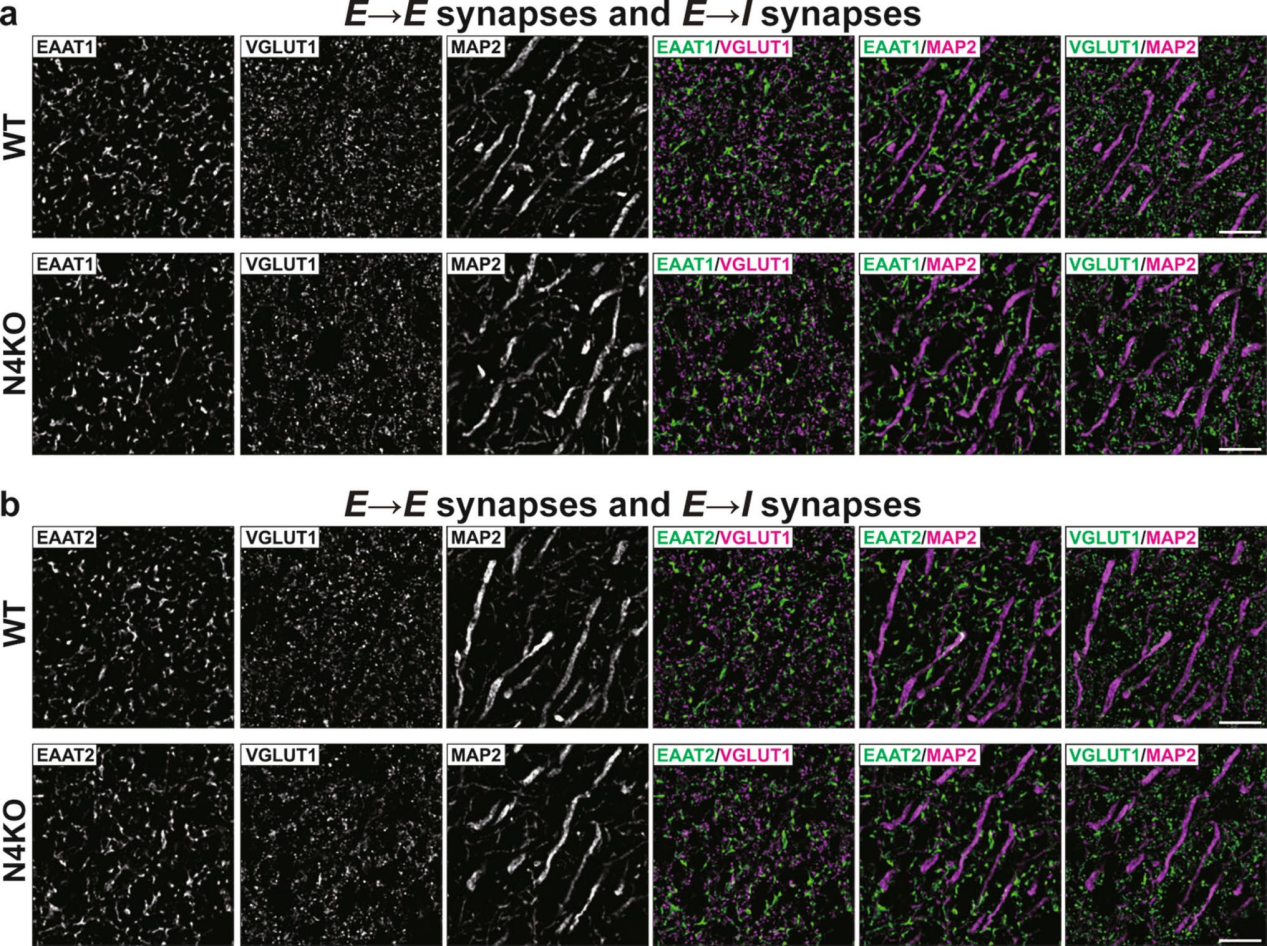
Synaptic degenerations in the *Necl-4*-knockout (N4KO) hippocampus. Representative images of hippocampi of wild-type (WT) and N4KO mice at 19 weeks of age using the indicated antibodies in respective panels. (a, b) Super-resolution structured illumination microscopy images of the *stratum radiatum* of CA1 region. Bars, 5 μm. These images are representative of three independent experiments.

In hippocampi of *Necl-4*-KO mice at 19 wk, the number of DAPI-positive cells, mainly consisting of pyramidal cells and a few inhibitory neurons and astrocytes, decreased, particularly at the *stratum pyramidale* of CA1, and granule cells also decreased at the *stratum granulosum* of DG, compared to those of hippocampi of WT mice at 19 wk (Fig. 3a). The respective numbers of PV-positive and calretinin-positive inhibitory neurons in CA1 also decreased (Fig. 3b, c, Table 1). In all *Necl-4*-KO hippocampal regions, the VGAT signal markedly decreased, while the VGLUT1 signal showed only a weak decrease. Meanwhile, the gephyrin and homer-1 signals remained unchanged (Fig. 3d–g). Areas for the GFAP, EAAT1/2, and GAT-3 signals decreased in all *Necl-4*-KO hippocampal regions, especially at CA1, compared to those in all WT hippocampal regions (Fig. 3h–k, Table 1), indicating that the astrocyte domain decreased in *Necl-4*-KO hippocampi. In SIM images of *I* → *E* synapses on pyramidal cells at the *stratum pyramidale* of CA1, the GAT-3 and VGAT signals, which localized nearby, were observed near Ctip2-positive nuclei in both WT and *Necl-4*-KO hippocampi, but the area without GAT-3 and VGAT signals was larger in *Necl-4*-KO hippocampi than in WT hippocampi (Fig. 4a, Table 1). These results suggest that *I* → *E* synapses are not properly distributed, and their numbers decreased, although *I* → *E* synapses are properly interacted with PAPs. In SIM images of *I* → *I* synapses on PV-positive dendritic shafts of inhibitory neurons at the *stratum lucidum* of CA3 near DG, the GAT-3 and VGAT signals localized nearby along PV-positive dendritic shafts in both WT and *Necl-4*-KO hippocampi, and their respective numbers in *Necl-4*-KO hippocampi were the same as those in WT hippocampi (Fig. 4b), suggesting that *I* → *I* synapses are properly formed without a reduction in their number and interacted with PAPs. In SIM images of the *stratum radiatum* of CA1, EAAT1/2-positive PAPs localized near VGLUT1-positive synapses, which include mostly *E* → *E* synapses and some *E* → *I* synapses, and these three signals were observed near MAP2-positive dendritic shafts in both WT and *Necl-4*-KO hippocampi (Fig. 5a, b). The VGLUT1 signal that was not observed on dendritic shafts may be derived from *E* → *E* and *E* → *I* synapses localized at dendritic branches. These synapses were uniformly distributed in WT hippocampi, but in *Necl-4*-KO hippocampi, there were regions where these synapses were absent (Fig. 5a, b). These results revealed that the respective numbers of *E* → *E* and *E* → *I* synapses at dendritic branches decrease in *Necl-4*-KO hippocampi, compared to those in WT hippocampi. Collectively, these results indicate that *Necl-4* genetic ablation induces both neuronal loss with synaptic degenerations and astrocytic degenerations in the hippocampus, implying that neuronal and/or astrocytic Necl-4 prevent(s) these neuronal and astrocytic impairments in the hippocampus.

### Synaptic Degenerations and Neuronal Death Through a Synaptic Cascade in Cultured *Necl-4*-KO Hippocampal Neurons

**Fig. 6:**
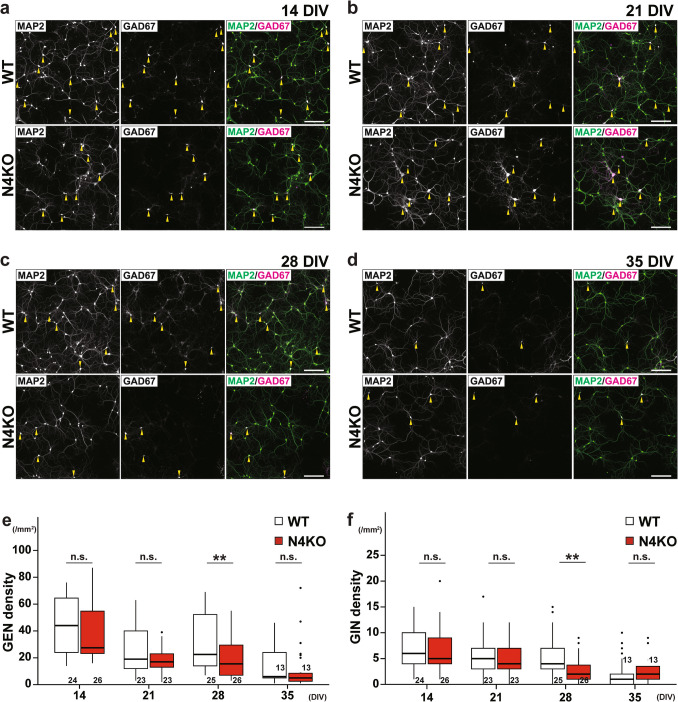
Time-dependent neuronal death in cultured *Necl-4*-knockout (N4KO) hippocampal neurons. Representative immunofluorescence images using the indicated antibodies in respective panels. (a–d) Cultured wild-type (WT) and N4KO hippocampal neurons at 14 (a), 21 (b), 28 (c), and 35 days in vitro (DIV) (d). Yellow arrowheads, GABAergic inhibitory neurons (GINs). Bars, 200 μm. These images are representative of three independent experiments. (e, f) Quantitative analysis of glutamatergic excitatory neuron (GEN) (e) and GIN (f) densities. **, p < 0.01. n.s., not significant. Two-tailed, unpaired Student’s *t*-tests were performed. Sample sizes are indicated within each boxplot.

To determine whether the synaptic degenerations and neuronal loss observed in *Necl-4*-KO hippocampi were caused by a deficiency of neuronal Necl-4, *Necl-4-*KO and WT hippocampal neurons were cultured for different periods, and the phenotypes of excitatory neurons, inhibitory neurons, and four types of synapses were compared using specific neuronal and synaptic markers. Synaptic degenerations and neuronal death were observed to a greater extent in *Necl-4-*KO neurons than in WT neurons after prolonged culture. Excitatory and inhibitory neurons died more rapidly in *Necl-4-*KO neurons than in WT neurons (Fig. 6a–f). The densities of excitatory and inhibitory neurons in *Necl-4-*KO neurons were not significantly different from those in WT neurons at 14 days in vitro (DIV) (Fig. 6a, e, f). However, the density of excitatory neurons, but not that of inhibitory neurons, decreased in *Necl-4-*KO neurons compared to WT neurons at 21 DIV, although this change was not significant (Fig. 6b, e, f). At 28 DIV, the densities of both excitatory and inhibitory neurons decreased in *Necl-4-*KO neurons compared to WT neurons (Fig. 6c, e, f). The decreases in the densities of both excitatory and inhibitory neurons were more pronounced in *Necl-4-*KO neurons, although a decrease was also observed in WT neurons at 35 DIV (Fig. 6d, e, f). These results indicate that *Necl-4* genetic ablation induces neuronal death in culture in a time-dependent manner, with excitatory neurons being the first to die, followed by inhibitory neurons.

**Fig. 7:**
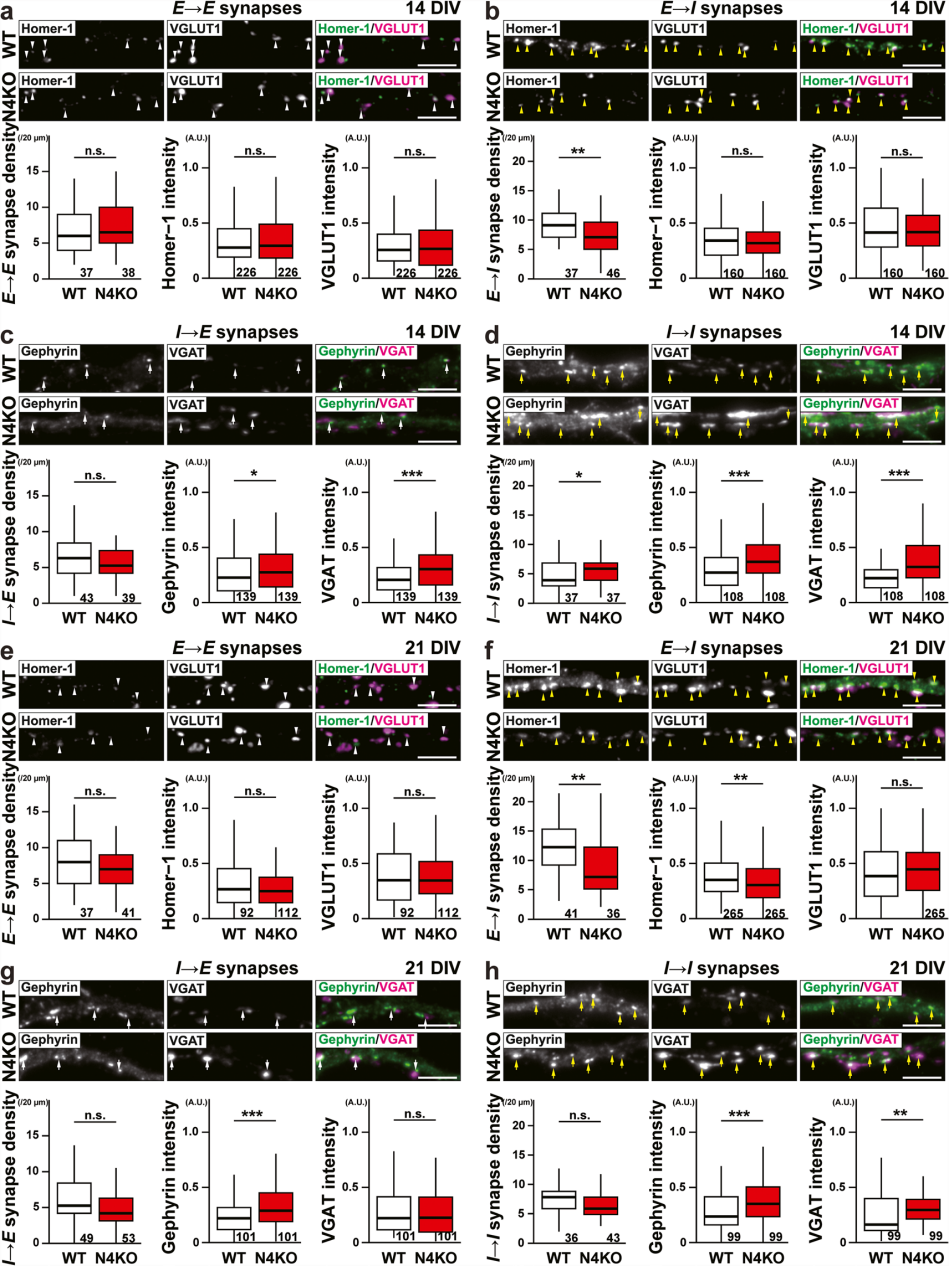
Time-dependent synaptic degenerations in cultured *Necl-4*-knockout (N4KO) hippocampal neurons. Representative immunofluorescence images using the indicated antibodies in respective panels. (a–l) High-magnification images of a dendrite of an excitatory neuron for glutamatergic and GABAergic synapses on that neuron (a, c, e, g, i, k; *E* → *E* and *I* → *E* synapses), and of a dendrite of an inhibitory neuron for glutamatergic and GABAergic synapses on that neuron (b, d, f, h, j, l; *I* → *E* and *I* → *I* synapses), in cultured wild-type (WT) and N4KO hippocampal neurons at 14 (a–d), 21 (e–h), and 28 days in vitro (DIV) (i–l). White arrowheads, *E* → *E* synapses. Yellow arrowheads, *E* → *I* synapses. White arrows, *I* → *E* synapses. Yellow arrows, *I* → *I* synapses. Bars, 5 μm. These images are representative of three independent experiments. Quantitative analyses for each synaptic marker are shown in the lower panels. *, p < 0.05, **, p < 0.01, ***, p < 0.001. n.s., not significant. Two-tailed, unpaired Student’s *t*-tests were performed. Sample sizes are indicated within each boxplot.

Phenotypic changes of synapses in cultured *Necl-4-*KO neurons before and after *Necl-4-*KO neuron death were analyzed. In *Necl-4-*KO neurons at 14 DIV, an increase in *I* → *I* synapse density, increases in gephyrin and VGAT signal intensities at *I* → *E* and *I* → *I* synapses (Fig. 7c, d), and a decrease in *E* → *I* synapse density were observed (Fig. 7b), but no phenotypic changes were observed at *E* → *E* synapses, compared to those in WT neurons (Fig. 7a). Most of the phenotypic changes of *I* → *I* synapses were observed in surviving *Necl-4* neurons at 21 DIV, although *I* → *I* synapse density was not significantly different from that of WT neurons (Fig. 7h). The increase in gephyrin signal intensity at *I* → *I* synapses was still observed in surviving *Necl-4*-KO neurons at 28 DIV, whereas the VGAT signal intensity at *I* → *I* synapses was not significantly different from that of WT neurons. However, *I* → *I* synapse density reverted to normal levels (Fig. 7l). The increase in gephyrin signal intensity at *I* → *E* synapses was also observed in surviving *Necl-4-*KO neurons at 21 and 28 DIVs (Fig. 7g, k). A decrease in homer-1 signal intensity at *E* → *I* synapse, in addition to the decrease in *E* → *I* synapse density, was observed in surviving *Necl-4-*KO neurons at 21 DIV, but not at 28 DIV (Fig. 7f, j). In addition, an increase in VGLUT1 signal intensity at *E* → *I* synapses was observed in *Necl-4-*KO neurons at 28 DIV (Fig. 7j). In contrast, no phenotypic changes of *E* → *E* synapses were observed in surviving *Necl-4-*KO neurons at 14, 21, or 28 DIV (Fig. 7a, e, i). According to these results, *Necl-4* genetic ablation induced synaptic degenerations through a synaptic cascade that lead to neuronal death in cultured hippocampal neurons. Considering the predominant Necl-4 localization at *I* → *I* synapses, the changes commenced at *I* → *I* synapses and spread to *E* → *I* and *I* → *E*, and *E* → *E* synapses in this sequence (Fig. 12a1), although there may have been cases in which *E* → *E* synapses were lost before phenotypic changes were detected. Taken together, these in vivo and in vitro results suggest that Necl-4 in inhibitory neurons regulates synaptic degenerations and neuronal death in the hippocampus.

### Synaptic GABA_A_R Upregulation at *I* → *I* Synapses in Cultured *Necl-4*-KO Hippocampal Neurons

**Fig. 8:**
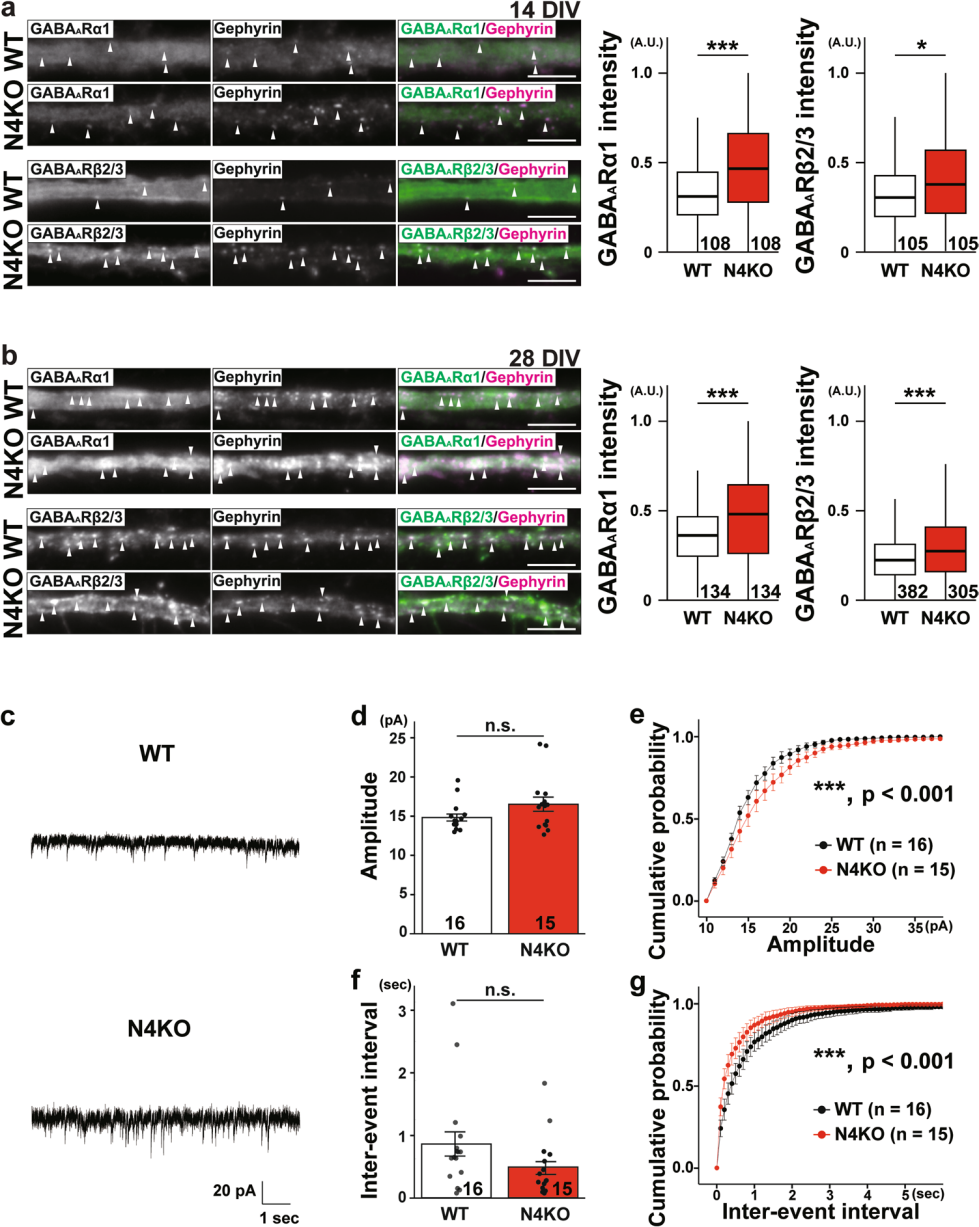
sGABA_A_R upregulation at *I* → *I* synapses in cultured *Necl-4*-knockout (N4KO) hippocampal neurons. Representative immunofluorescence images using the indicated antibodies in respective panels. (a) High-magnification images of a dendrite of an inhibitory neuron from cultured wild-type (WT) and N4KO hippocampal neurons at 14 days in vitro (DIV). White arrowheads, GABAergic synapses on inhibitory neurons (*I* → *I* synapses). (b) High-magnification images of a dendrite of an inhibitory neuron in cultured WT and N4KO neurons at 28 DIV. White arrowheads, *I* → *I* synapses. Bars, 5 μm. These images are representative of three independent experiments. Quantitative analyses are shown in the right panels. *, p < 0.05. ***, p < 0.001. Two-tailed, unpaired Student’s *t*-tests were performed. (c) Representative traces of miniature inhibitory postsynaptic currents (mIPSCs) recorded from inhibitory neurons in cultured WT and N4KO hippocampal neurons at 23–25 DIV. (d, f) Summary graphs showing the mean amplitude (d) and inter-event interval (f) of mIPSCs recorded from inhibitory neurons (e, g) Cumulative probability plots of mIPSC amplitude (e) and inter-event interval (g) from the same cells as in (d, f). For comparisons of mean amplitude and inter-event interval (d, f), two-tailed, unpaired Student’s *t*-tests were performed with a Bonferroni correction. For cumulative probability analyses (e, g), Kolmogorov–Smirnov tests were used. ***, adjusted p < 0.001. n.s., not significant. Sample sizes are indicated within each graph. GABA_A_R α1 subunit (GABA_A_Rα1). GABA_A_R β2/3 subunit (GABA_A_Rβ2/3).

We next examined whether *Necl-4*-KO also affects GABA_A_Rs at *I* → *I* synapses. There are two types of GABA_A_Rs: synaptic GABA_A_R (sGABA_A_R), which localizes at the synaptic region and regulates phasic inhibition, and perisynaptic GABA_A_R (pGABA_A_R), which localizes at the perisynaptic region and regulates tonic inhibition [35]. GABA_A_R α1 subunit is known to localize at the synaptic region and is referred to as sGABA_A_R, whereas GABA_A_R β2/3 subunit is known to localize both at synaptic regions and perisynaptic regions and is referred to as s/pGABA_A_R. At *I* → *I* synapses in cultured hippocampal WT neurons at 14 DIV, the GABA_A_R α1 subunit signals were weak and faint (Fig. 8a). At *I* → *I* synapses in cultured WT neurons at 21 DIV, the Necl-4 signal co-localized with the GABA_A_R α1 subunit signal (Fig. 1h). However, at *I* → *I* synapses in cultured *Necl-4-*KO neurons at 14 DIV, the GABA_A_R α1 subunit and the GABA_A_R β2/3 subunit signals increased compared to those in WT neurons, and mainly localized near the gephyrin signal (Fig. 8a). These phenotypes were also observed at 28 DIV (Fig. 8b). The properties of *I* → *I* synapses were next examined by electrophysiological recordings in cultured hippocampal neurons. Recordings were performed from typical inhibitory neurons identified based on their morphological features. There were no significant differences in the mean amplitude or frequency of mIPSCs between WT and *Necl-4*-KO neurons (Fig. 8c, d, f). However, significant differences were detected in the cumulative probabilities of these parameters (Fig. 8c, e, g). The cumulative probability of mIPSC amplitudes in *Necl-4*-KO neurons was higher than in control WT neurons, suggesting an enhanced postsynaptic response to GABA in *Necl-4*-KO neurons (Fig. 8e). In addition, the cumulative probability of mIPSC inter-event intervals in cultured *Necl-4*-KO neurons was lower than in control WT neurons, possibly reflecting an increased number of *I* → *I* synapses or a higher probability of GABA release at *I* → *I* synapses (Fig. 8g). These results indicate that Necl-4 suppresses sGABA_A_R upregulation at *I* → *I* synapses and that *Necl-4*-KO alters inhibitory neurotransmission in a synapse-type specific manner in cultured hippocampal neurons (Fig. 12b).

### sGABA_A_R Upregulation by ErbB4 Activation at *I → I* Synapses in Cultured *Necl-4*-KO Hippocampal Neurons

**Fig. 9:**
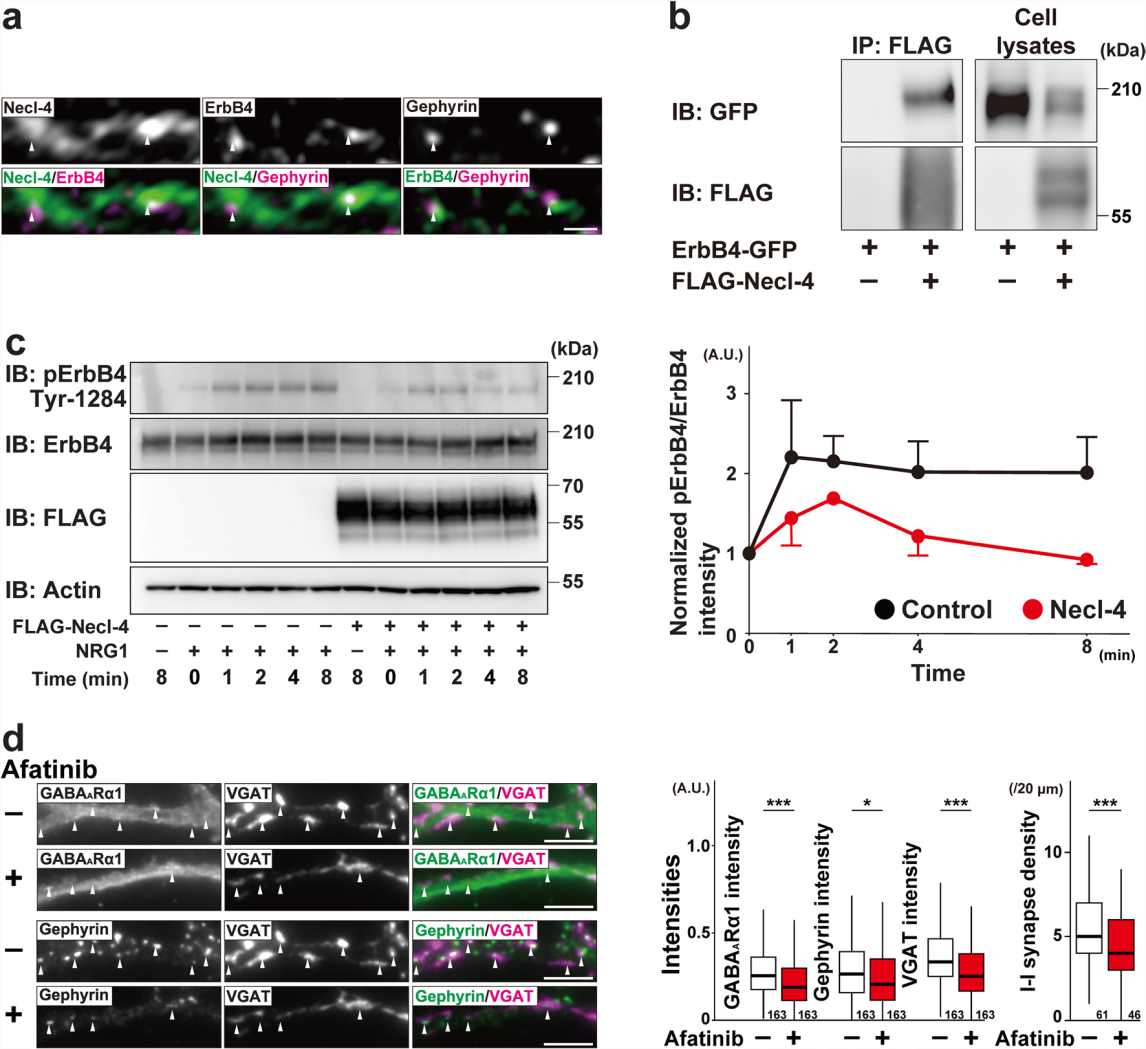
sGABA_A_R upregulation by ErbB4 activation at *I* → *I* synapses in cultured *Necl-4*-knockout (KO) hippocampal neurons. Representative images using the indicated antibodies (Abs) in respective panels. (a) Super-resolution structured illumination microscopy images of a dendrite of an inhibitory neuron in cultured wild-type (WT) hippocampal neurons at 21 days in vitro (DIV). Bar, 500 nm. White arrowheads, Necl-4 puncta colocalizing with gephyrin at GABAergic synapses on inhibitory neurons (*I* → *I* synapses). (b) HEK293E cells were co-transfected with a combination of the indicated plasmids. FLAG-Necl-4 was immunoprecipitated using the anti-FLAG monoclonal antibody, and samples were subjected to SDS-PAGE followed by Western blotting using the indicated Abs. The uncropped blot images are shown in Fig. S3a. (c) Control or Necl-4 stably expressed T47D cells were incubated with 20 ng/mL of neuregulin-1 (NRG1) for 1, 2, 4, and 8 min. Samples were subjected to SDS-PAGE followed by Western blotting using the indicated Abs. The uncropped blot images are shown in Fig. S3b. Quantitative analyses are shown in the right panels. Band intensities of phospho-ErbB4 on Tyr-1284 were normalized to that of total ErbB4 protein, and the normalized value of bulk control cells treated with NRG1 was set as 1.0. All data are presented as mean and standard error of the mean. (d) High-magnification images of a dendrite of an inhibitory neuron in cultured *Necl-4*-KO neurons at 14 DIV. Cultured N4KO neurons were incubated in the presence or absence of 100 nM of afatinib for 48 h from 12 DIV and then fixed at 14 DIV. Bars, 5 μm. White arrowheads, *I* → *I* synapses. These images are representative of three independent experiments. Quantitative analyses are shown in the right panels. *, p < 0.05, ***, p < 0.001. Two-tailed, unpaired Student’s *t*-tests were performed. Sample sizes are indicated within each boxplot.

It was previously shown that overexpression of ErbB4 increases presynaptic inputs at *E* → *I* and *I* → *I* synapses [36], that Necl-2 *cis*-interacts with ErbB4 and inhibits NRG1-induced ErbB4 activation at *E* → *I* synapses [37], and that NRG1 induces GABA_A_R expression in cultured rat cerebellar granule cells [38]. Consistently, in cultured WT hippocampal neurons at 21 DIV, the Necl-4 signal co-localized with the ErbB4 signal at the flanking region of the gephyrin signal at *I* → *I* synapses in cultured WT hippocampal neurons at 21 DIV, as revealed by SIM imaging (Fig. 9a). To examine the potential interaction between Necl-4 and ErbB4, immunoprecipitation experiments were performed using HEK293E cells, which do not endogenously express either Necl-4 or ErbB4. When FLAG-Necl-4 was immunoprecipitated using an anti-FLAG mAb, ErbB4-GFP was co-immunoprecipitated with FLAG-Necl-4 (Fig. 9b). Next, to investigate whether Necl-4 affects ErbB4 activation, T47D cells stably expressing FLAG-Necl-4 were treated with NRG1 for various periods of time. Although T47D cells do not endogenously express Necl-4, they do express ErbB4 [28]. In control T47D cells, ErbB4 was phosphorylated at Tyr-1284 one minute after NRG1 application, and this phosphorylation continued to increase at least 8 min (Fig. 9c). However, in T47D cells stably expressing Necl-4, the phosphorylation decreased (Fig. 9c). Afatinib, an ErbB1/2/4 inhibitor, reduced the elevated GABA_A_R α1 subunit, gephyrin, and VGAT signals at *I* → *I* synapses and elevated *I* → *I* synapse density in cultured *Necl-4-*KO neurons at 14 DIV (Fig. 9d). These results indicate that Necl-4 *cis*-interacts with and inactivates ErbB4, which suppresses the upregulation of both sGABA_A_R and gephyrin at *I* → *I* synapses, and the increase in *I* → *I* synapse density in cultured hippocampal neurons (Fig. 12a2, b).

### E/I balance Disorders in Cultured *Necl-4*-KO Hippocampal Neurons

**Fig. 10:**
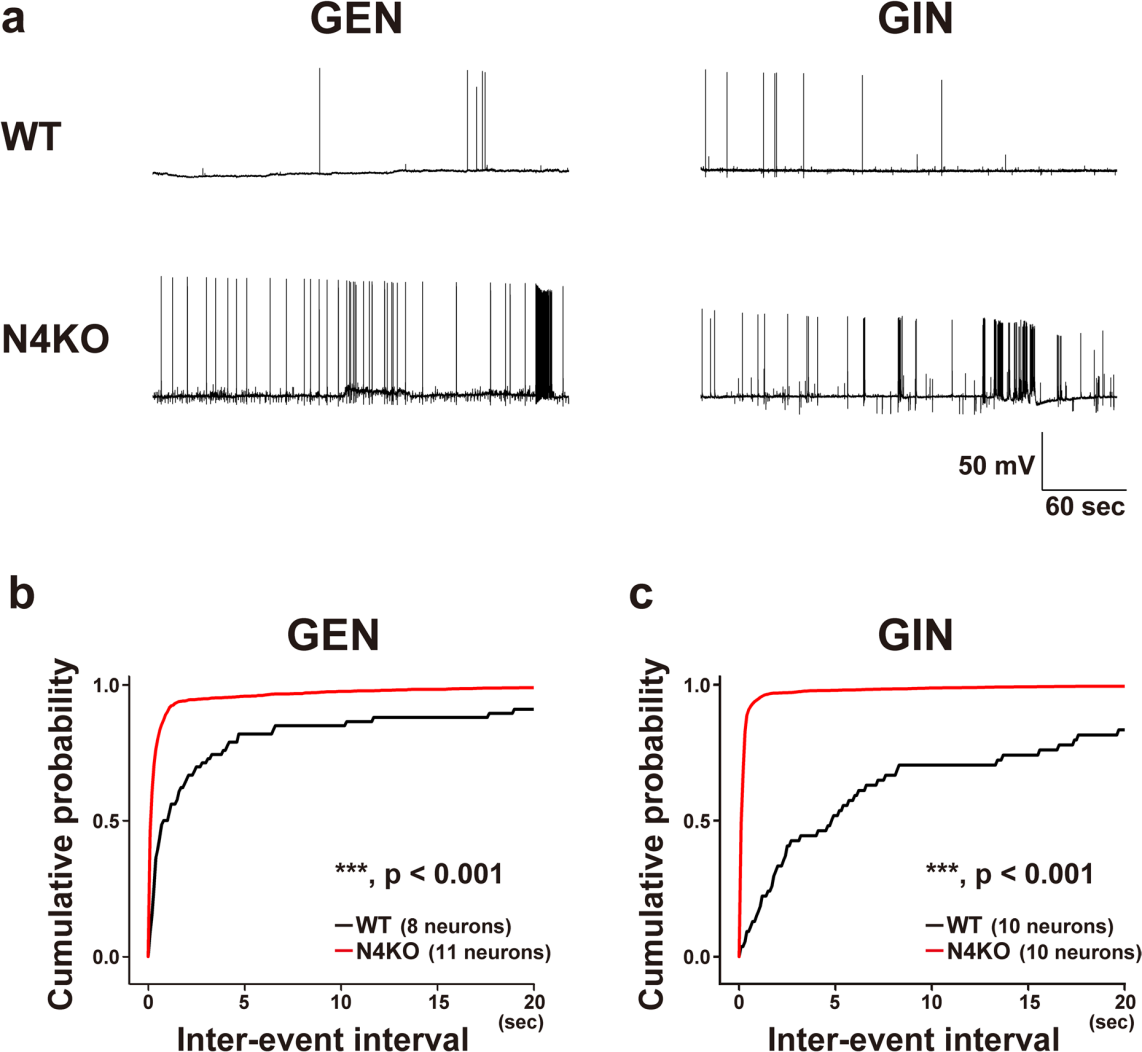
Electrophysiological analysis of spontaneous firing in cultured excitatory and inhibitory neurons from wild-type (WT) and *Necl-4*-knockout (N4KO) mice. (a) Membrane potentials recorded from glutamatergic excitatory neurons (GENs) and GABAergic inhibitory neurons (GINs) in cultured WT (upper) and N4KO hippocampal neurons (lower) at 23–25 days in vitro. (b, c) Cumulative probabilities of inter-event intervals of firing recorded from GENs (b) (WT, 66 events; N4KO, 2,059 events) and GINs (c) (WT, 54 events; N4KO, 3,966 events) in cultured WT and N4KO neurons. In (b) and (c), cumulative probability plots were generated using the data from the subset of recorded neurons that exhibited two or more spontaneous action potentials during the 5-min recording period. For cumulative probability analyses (b, c), Kolmogorov–Smirnov tests were used. ***, adjusted p < 0.001. n.s., not significant. Sample sizes are indicated within each graph.

Suppression of phasic inhibition by sGABA_A_R upregulation at *I* → *I* synapses may elicit abnormal electrophysiological properties of excitatory and inhibitory neurons in cultured *Necl-4*-KO hippocampal neurons. To address this possibility, we measured the membrane potentials of excitatory and inhibitory neurons and examined spontaneous action potentials (sAPs). Resting membrane potentials of excitatory and inhibitory neurons did not differ significantly between cultured WT and *Necl-4*-KO neurons at either 14–16 DIV or 23–25 DIV. At 14–16 DIV, the resting membrane potentials of excitatory neurons were −76.80 ± 3.26 mV (WT, 7 cells) and −74.19 ± 1.91 mV (*Necl-4*-KO, 8 cells; p = 0.488, two-tailed, unpaired Student’s *t*-test), while those of inhibitory neurons were −68.52 ± 2.52 mV (WT, 7 cells) and −67.20 ± 2.43 mV (*Necl-4*-KO, 10 cells; p = 0.354, two-tailed, unpaired Student’s *t*-test). At 23–25 DIV, the resting membrane potentials of excitatory neurons were −73.87 ± 1.58 mV (WT, 27 cells) and −68.79 ± 2.31 mV (*Necl-4*-KO, 23 cells; p = 0.0694, two-tailed, unpaired Student’s *t*-test), while those of inhibitory neurons were −72.52 ± 1.32 mV (WT, 24 cells) and −70.58 ± 1.40 mV (*Necl-4*-KO, 25 cells; p = 0.320, two-tailed, unpaired Student’s *t*-test). Neither excitatory nor inhibitory neurons in WT and *Necl-4*-KO neurons cultured at 14–16 DIV showed more than two sAP firings during a 5-min membrane potential recording. In 23–25 DIV, two or more action potentials were recorded in approximately 30–40% of cells during a 5-min membrane potential recording (Fig. 10a). However, in cultured *Necl-4*-KO neurons, both excitatory and inhibitory neurons repeatedly showed high-frequency sAP firings with short inter-event intervals, whereas such high-frequency burst-like firings were not observed in cultured WT neurons (Fig. 10a–c). There was no significant difference between WT and *Necl-4*-KO neurons in the percentage of cells that elicited two or more action potentials in 5 min (excitatory neurons: WT, 8 of 27 cells; *Necl-4*-KO, 11 of 23 cells; inhibitory neurons: WT, 10 of 24 cells; *Necl-4*-KO, 10 of 25 cells). However, among the *Necl-4*-KO neurons in which two or more action potentials were observed, most excitatory and inhibitory neurons showed repeated high-frequency firings with short inter-event intervals. Such firing patterns were not observed in cultured WT neurons (Fig. 10a–c). These results suggest that Necl-4 prevents excessive firing in a subset of excitatory and inhibitory neurons (Fig. 12a2, b). The exact reason why only a small population of excitatory and inhibitory neurons showed high-frequency firing is not known, but it may be related to the formation of multiple neural network subsets within the cultured *Necl-4*-KO neuronal networks used in this study.

### Involvement of Excitotoxicity in Neuronal Death in Cultured *Necl-4*-KO Hippocampal Neurons

**Fig. 11:**
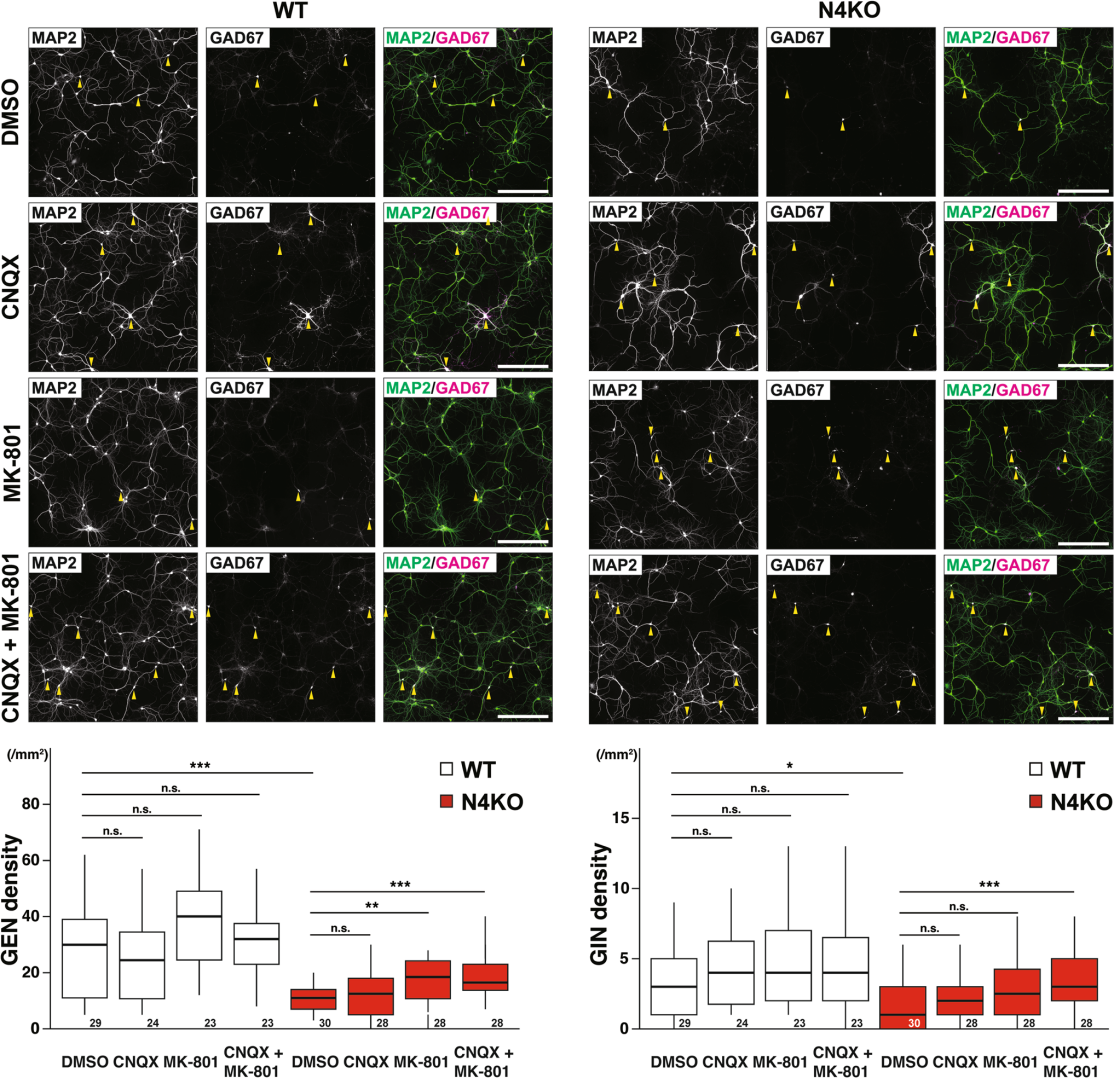
Involvement of excitotoxicity in neuronal death in cultured *Necl-4*-knockout (N4KO) hippocampal neurons. Representative immunofluorescence images using the indicated antibodies in respective panels. Cultured wild-type (WT) and N4KO hippocampal neurons at 28 days in vitro (DIV). Cultured neurons were incubated in the presence or absence of 10 μM of 6-cyano-7-nitroquinoxaline-2,3-dione (CNQX) and 20 μM of MK-801 from 14 DIV, and then fixed at 28 DIV. Yellow arrowheads, GABAergic inhibitory neurons (GINs). Bars, 300 μm. These images are representative of three independent experiments. Quantitative analyses are shown in the lower panels. *, adjusted p < 0.05. **, adjusted p < 0.01. ***, adjusted p < 0.001. n.s., not significant. Two-tailed, unpaired Student’s *t*-tests were performed with a Bonferroni correction. Sample sizes are indicated within each boxplot.

High-frequency stimulation that induces long-term potentiation (LTP) of excitatory neurons leads to the excessive glutamate release at their axon terminals [39]. The expression and localization of AMPA receptors are tightly regulated during LTP [40]. Excessive glutamate release causes excitotoxicity through extra- or perisynaptic NMDA receptor (e/pNMDAR) [41, 42]. To test whether such excitotoxic mechanisms are involved in the neuronal death observed in our culture model, we pharmacologically inhibited glutamate receptors in cultured *Necl-4*-KO hippocampal neurons. We used MK-801, an NMDA receptor antagonist, to block NMDA receptor-mediated excitotoxicity, and CNQX, an AMPA receptor antagonist, to examine whether AMPA receptor-mediated mechanisms also contribute to the neuronal death. Death of excitatory neurons observed in cultured *Necl-4*-KO hippocampal neurons was suppressed by treatment with MK-801 either alone or in combination with CNQX, but not with CNQX alone (Fig. 11). Death of inhibitory neurons was suppressed by treatment with a combination of CNQX and MK-801, but not with MK-801 alone or CNQX alone (Fig. 11). These results indicate that neuronal death observed in cultured *Necl-4-*KO hippocampal neurons is at least partly mediated by excitotoxicity through e/pNMDAR at glutamatergic synapses.

## Discussion

**Fig. 12:**
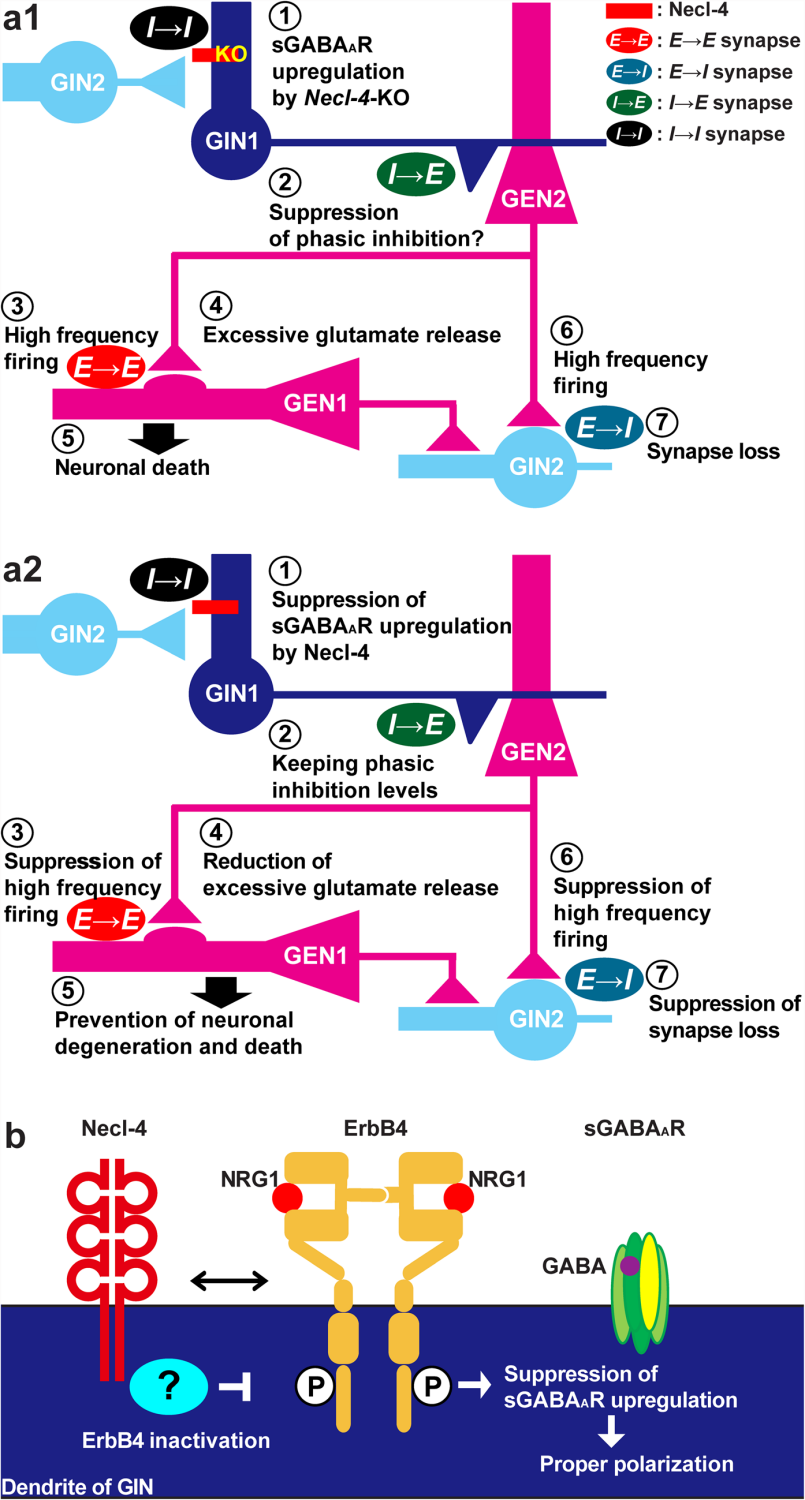
Regulatory mechanisms of E/I balance by Necl-4 at *I* → *I* synapses in cultured hippocampal neurons. (a1) Model of mechanisms of excitation and inhibition balance (E/I balance) disorders leading to synaptic degenerations and neuronal death by *Necl-4*-knockout (N4KO) at GABAergic synapses on GABAergic inhibitory neurons (GINs) (*I* → *I* synapses). ① *Necl-4* genetic ablation at *I* → *I* synapses on a dendrite of GIN1 induces synaptic GABA_A_ receptor (sGABA_A_R) upregulation, ② probably suppressing phasic inhibition of GIN1. ③ This results in high-frequency firing of glutamatergic excitatory neuron (GEN) 2, ④ consequently releasing excessive glutamate from glutamatergic synapses on GEN1 (*E* → *E* synapses) and ⑤ leading to neuronal death, whereas ⑥ high-frequency firing of GEN2 causes high-frequency firing of GIN2 ⑦ lead to synapse loss of glutamatergic synapses on GIN2 (*E* → *I* synapses). (a2) Model of mechanisms of E/I balance regulation by Necl-4 at *I* → *I* synapses to ensure proper neural network activity. ① Necl-4 at *I* → *I* synapses on a dendrite of GIN1 suppresses sGABA_A_R upregulation, ② maintaining proper phasic inhibition levels of GIN1. ③ This suppresses high-frequency firing of GEN2, ④ consequently reducing excessive glutamate release from *E* → *E* synapses and ⑤ preventing neuronal degenerations and neuronal death, whereas ⑥ suppressed high-frequency firing of GEN2 suppresses high-frequency firing of GIN2 ⑦ lead to suppression of synapse loss of *E* → *I* synapses. (b) Mechanisms to control sGABA_A_R-mediated polarization levels by Necl-4 via ErbB4 inactivation at *I* → *I* synapses. Necl-4 inhibits neuregulin-1 (NRG1)-induced ErbB4 activation through *cis*-interaction.

We showed here the localization of Necl-4 in neurons, especially inhibitory neurons, in cultured mouse hippocampal neurons and in the mouse hippocampus in addition to astrocytes and oligodendrocytes. Our detailed studies on Necl-4 using cultured WT and *Necl-4*-KO neurons revealed that Necl-4 contributes to the regulation of inhibitory neuronal functions, thereby influencing the E/I balance in our in vitro model of cultured hippocampal neurons. This regulation is achieved by downregulating sGABA_A_R via ErbB4 inactivation at *I* → *I* synapses and/or by decreasing *E* → *I* synapse density. These mechanisms inhibit high-frequency firing of local neural networks comprising excitatory and inhibitory neurons, thereby preventing excessive glutamate release (Fig. 12a2, b). In contrast to the well-established functions of glutamatergic *E* → *E* and *E* → *I* synapses in relation to neuronal impairments, the functions of GABAergic *I* → *E* and *I* → *I* synapses remain largely unclear. This study reveals a novel regulatory mechanism of inhibitory neurons by which Necl-4 suppresses overexcitability through phasic inhibition in neural network activity. It also suggests the importance of *I* → *I* synapse function in controlling entire neural network activity. We also found that neuronal loss, including that of excitatory and inhibitory neurons, occurred in *Necl-4*-KO hippocampi in vivo, and this loss is likely caused by neuronal death, as neuronal death induced by excitotoxicity through NMDAR was observed in the *Necl-4*-KO hippocampal neuron cultures in vitro. These results indicate that the neuronal death is regulated at least partly by neuronal Necl-4.

Consistent with these findings, we observed that neuronal death in *Necl-4*-KO neurons became prominent at later time points in vitro, for example, around 21 DIV, whereas minimal cell death was detected at earlier stages such as 14 DIV. These results suggest that the loss of Necl-4 leads to a progressive deterioration of neuronal viability over time. This time-dependent progression may reflect the cumulative impact of dysregulated inhibitory synaptic input onto inhibitory neurons, leading to hyperexcitability in excitatory neurons and disruption of the E/I balance. In particular, the increased density of GABAergic synapses and high-frequency firing observed in *Necl-4*-KO neurons likely contribute to chronic network instability and excitotoxicity, ultimately resulting in neuronal loss. Although the precise physiological consequences of this time-dependent neuronal death remain to be fully elucidated, similar mechanisms could underlie neuronal vulnerability during aging or neurodegenerative diseases characterized by progressive neuronal loss and altered E/I balance. Further studies will be necessary to elucidate the potential links in greater detail.

In vitro experiments using cultured hippocampal neurons showed that *Necl-4* genetic ablation resulted in phenotypic changes at *E* → *I*, *I* → *E*, and *I* → *I* synapses. The predominant localization of Necl-4 at *I* → *I* synapses, rather than *E* → *I* and *I* → *E* synapses, suggests that the synaptic degenerations and neuronal death originate from *I* → *I* synapses, although it is possible that degenerations of *E* → *I* and *I* → *E* synapses are directly and initially caused by *Necl-4* genetic ablation. It was previously reported that Necl-4 is identified at purified synaptic plasma membranes from the rat forebrain, implying an important role for Necl-4 at synapses [43]. It was also reported that in vivo chemico-genetic proximity-labeling techniques identified Necl-4 as being localized near collybistin at inhibitory synapses [44]. These previous reports did not differentiate between synapse types, but together with our results, *I* → *I* synapses may play a crucial role in Necl-4 function and its associated disorders. Further investigation is needed to determine which synapses—*I* → *I* and/or others—are essential for preventing synaptic degeneration and neuronal impairments. In contrast, no phenotypic changes of *E* → *E* synapses were detected in surviving *Necl-4*-KO neurons in our in vitro study, which might be due to the rapid loss of *E* → *E* synapses. Our findings that the decreases in the respective numbers of *E* → *E* and *E* → *I* synapses at dendritic branches in *Necl-4*-KO hippocampi, along with the previous finding that the decreases in excitatory synaptic molecules in postsynaptic density fraction of *Necl-4*-KO hippocampi [45], consistently indicate that *Necl-4* genetic ablation induces changes in excitatory synapses in vivo, although it remains to be determined whether these changes are direct or secondary effects. In any case, these results indicate that Necl-4 in inhibitory neurons regulates the E/I balance of the entire neural network activity, thereby preventing synaptic degenerations and neuronal death caused by excitotoxicity.

Synaptic degenerations observed in cultured *Necl-4*-KO hippocampal neurons in vitro, such as *I* → *E* synapse degenerations, were also observed in the *Necl-4*-KO hippocampus in vivo, but changes in *I* → *I* and *I* → *E* synapses in vitro differed from those observed in vivo, and *E* → *E* synapse degenerations and astrocyte domain decrease were additionally observed in vivo. These different abnormal phenotypes in vivo may occur due to the expression of Necl-4 not only in inhibitory neurons but also in astrocytes and oligodendrocytes. For instance, Necl-4 is expressed in oligodendrocytes and involved in myelination in the white matter [34, 46], and Necl-4 regulates adhesion between Schwann cells and dorsal root ganglion axons and between Schwann cells in Schmidt-Lanterman incisure in peripheral nerves [47, 48]. Our results indicate that Necl-4 in neurons plays an indispensable role in maintaining neuronal function, but further studies are necessary to understand the respective functions and modes of action of Necl-4 in inhibitory neurons and glia in neuronal impairments and neural network disorders.

Based on the results of the cytological and electrophysiological experiments, we illustrated a model in Fig. 12a, b. At *I* → *I* synapses, Necl-4 suppresses upregulation of sGABA_A_R at 14 DIV. In the absence of Necl-4, this suppression is lost, leading to activation of *I* → *I* synapses. Consequently, the activity of inhibitory neurons is expected to be suppressed, which in turn is likely to decrease *I* → *E* synapse function. However, the cytological examination showed an increase in *I* → *E* synapse molecules, suggesting increased *I* → *E* synapse activity. Based on the results that neuronal death in *Necl-4*-KO culture is at least partly mediated by excitotoxicity, the suppressive function of *I* → *E* synapses on the excitability of excitatory neurons is either insufficient or lost. Thus, *Necl-4*-KO inhibitory neurons may not suppress network excitability as effectively as WT inhibitory neurons. This results in high-frequency firing of excitatory neurons, as shown in the electrophysiological experiments, releasing excessive glutamate from *E* → *E* and *E* → *I* synapses at 23–25 DIV. Repeated glutamate release from *E* → *E* synapses further induces high-frequency firing in excitatory neurons, exciting them and leading to death of excitatory neurons. Similarly, glutamate release from *E* → *I* synapses also induces high-frequency firing in inhibitory neurons, potentially resulting in long-term depression and *E* → *I* synapse loss. One possible explanation for the difference between the cytological evidence of inhibitory neuron dysfunction and electrophysiological evidence of inhibitory neuron hyperactivation is the relative contribution of *E* → *I* and *I* → *I* synapses to the overall excitability of inhibitory neurons. In fact, the increase in *I* → *I* synapse density reverted to normal level, and the VGLUT1 signal intensity at *E* → *I* synapses increased at 28 DIV. This suggests that the balance of *E* → *I* and *I* → *I* synapses shifts to enhance the activity of inhibitory neurons as a secondary consequence of changes in the neural circuit. Another possible explanation for the difference is non-synaptic regulation, such as extra- or perisynaptic NMDARs [42]. It has been previously shown that NMDARs localized at extra- or perisynaptic regions, rather than synaptic regions, mediate tonic current and increase neuronal activity [49]. Repeated release of excessive glutamate at *E* → *I* synapses can stimulate these NMDARs, resulting in increased excitability of inhibitory neurons and subsequent increased *I* → *E* synapse activity. The reason why neuronal death was suppressed after *I* → *E* synapse activity increased may be due to the accumulation of glutamate or other secreted factors. Considering the predominant localization of Necl-4 at *I* → *I* synapses, dysregulation of *I* → *I* synapses due to *Necl-4* genetic ablation may contribute to the impairments of *E* → *I* and *I* → *E* synapses, as well as neuronal death signaling.

While our electrophysiological experiments provide important insights into the functional consequences of these synaptic alterations, it is important to consider several limitations of the experimental conditions. First, the experiments were conducted at room temperature, which is considerably lower than physiological temperature (~ 37ºC), due to technical constraints specific to whole-cell patch-clamp recordings in cultured neurons. Based on our prior experience, higher temperatures tend to make the neuronal membrane more fragile, making it difficult to maintain stable recordings for the extended periods (over five minutes) required in this study. Second, the experiments were conducted in a medium containing 30 mM glucose to maintain the health and viability of the cultured neurons. Higher glucose concentrations have been shown to support better cellular conditions and to slightly increase the frequency of action potentials compared to lower concentrations, ensuring more consistent and reliable data acquisition [50]. While these experimental conditions for obtaining stable recordings from cultured neurons differ from physiological settings and may affect neuronal excitability and synaptic activity to some extent, the overall trends observed are considered valid and provide meaningful insights into the underlying mechanisms. Nevertheless, future studies under more physiologically relevant conditions will be important to further validate and extend these findings.

The present results raise the possibility that Necl-4 expression levels regulate E/I balance, dysfunctions of which cause excitotoxicity. Neurons, astrocytes, and microglia secrete various cytokines and chemokines, which are neuroprotective and/or neurotoxic factors [51, 52]. Expression levels of some of these factors are altered by aging or AD [52]. In AD, synaptic degenerations and neuronal death are promoted by amyloid-β and astrocytic APOE4 [53]. In addition, decreased oxygen and glucose levels resulting from aging-dependent changes and pathogenicity may also contribute to synaptic degenerations and neuronal death [54]. Therefore, Necl-4 expression levels may be regulated by these physiological and/or pathological factors. E/I balance is well-controlled for brain development and functions, and its dysfunctions are implicated in aging-dependent neuronal impairments leading to various neurological diseases. Understanding the regulatory mechanisms of Necl-4 expression levels in vivo will provide insight into the roles of Necl-4 in physiological and pathological processes.

## Supplementary Information

Below is the link to the electronic supplementary material.

Supplementary file1 (TIFF 51264 KB)

Supplementary file2 (TIFF 33373 KB)

Supplementary file3 (TIFF 19921 KB)

## Data Availability

All data reported in this paper is available from the corresponding authors on reasonable request.

## Abbreviations

AD: Alzheimer’s disease
Abs: Antibodies
BSA: Bovine serum albumin
CA1: CA1 region
CA3: CA3 region
CNQX: 6-Cyano-7-nitroquinoxaline-2,3-dione
DG: Dentate gyrus
DIV: Days in vitro
DTT: Dithiothreitol
E/I: Excitation/inhibition
GABA: γ-Aminobutyric acid
GABAergic: γ-Aminobutyric acidergic
GABA_A_R: GABA_A_ receptor
pGABA_A_R: Perisynaptic GABA_A_R
sGABA_A_R: Synaptic GABA_A_R
HRP: Horseradish peroxidase
KO: Knockout
mAb: Monoclonal Ab
LTP: Long-term potentiation
MBP: Myelin basic protein
N4KO: *Necl-4*-KO
e/pNMDAR: Extra- or perisynaptic NMDA receptor
NRG1: Neuregulin-1
P0: Postnatal day 0
P56: Postnatal day 56
PAPs: Perisynaptic astrocyte processes
PBS: Phosphate-buffered saline
PFA: Paraformaldehyde
PV: Parvalbumin
PVA: Polyvinyl alcohol
ROIs: Regions of interest
RFP: Red fluorescent protein
sAPs: Spontaneous action potentials
sgRNA: Single-guide RNA
SIM: Super-resolution structured illumination microscopy
*E* → *E* synapses: Glutamatergic synapses on excitatory neurons
*E* → *I* synapses: Glutamatergic synapses on inhibitory neurons
*I* → *E* synapses: GABAergic synapses on excitatory neurons
*I* → *I* synapses: GABAergic synapses on inhibitory neurons
TBS: Tris-buffered saline
WT: Wild-type
19 wk: 19 Weeks of age
